# *Toxoplasma gondii* Dense Granule Proteins 7, 14, and 15 Are Involved in Modification and Control of the Immune Response Mediated via NF-κB Pathway

**DOI:** 10.3389/fimmu.2020.01709

**Published:** 2020-07-31

**Authors:** Fumiaki Ihara, Ragab M. Fereig, Yuu Himori, Kyohko Kameyama, Kosuke Umeda, Sachi Tanaka, Rina Ikeda, Masahiro Yamamoto, Yoshifumi Nishikawa

**Affiliations:** ^1^National Research Center for Protozoan Diseases, Obihiro University of Agriculture and Veterinary Medicine, Obihiro, Japan; ^2^Department of Animal Medicine, Faculty of Veterinary Medicine, South Valley University, Qena City, Egypt; ^3^Division of Animal Science, Department of Agricultural and Life Sciences, Faculty of Agriculture, Shinshu University, Nagano, Japan; ^4^Department of Immunoparasitology, Research Institute for Microbial Diseases, Osaka University, Osaka, Japan; ^5^Laboratory of Immunoparasitology, WPI Immunology Frontier Research Center, Osaka University, Osaka, Japan

**Keywords:** *Toxoplasma gondii*, dense granule protein, NFκB, immune response, host-pathogen interaction

## Abstract

*Toxoplasma gondii* infects almost all warm-blooded animals, including humans, leading to both cellular and humoral immune responses in the host. The virulence of *T. gondii* is strain specific and is defined by secreted effector proteins that disturb host immunity. Here, we focus on nuclear factor-kappa B (NFκB) signaling, which regulates the induction of T-helper type 1 immunity. A luciferase assay for screening effector proteins, including ROPs and GRAs that have biological activity against an NFκB-dependent reporter plasmid, found that overexpression of GRA7, 14, and 15 of a type II strain resulted in a strong activity. Thus, our study was aimed at understanding the involvement of NFκB in the pathogenesis of toxoplasmosis through a comparative analysis of these three molecules. We found that GRA7 and GRA14 were partially involved in the activation of NFκB, whereas GRA15 was essential for NFκB activation. The deletion of GRA7, GRA14, and GRA15 in the type II Prugniaud (Pru) strain resulted in a defect in the nuclear translocation of RelA. Cells infected with the PruΔgra15 parasite showed reduced phosphorylation of inhibitor-κBα. GRA7, GRA14, and GRA15 deficiency decreased the levels of interleukin-6 in RAW246.7 cells, and RNA-seq analysis revealed that GRA7, GRA14, and GRA15 deficiency predominantly resulted in downregulation of gene expression mediated by NFκB. The virulence of all mutant strains increased, but PruΔgra14 only showed a slight increase in virulence. However, the intra-footpad injection of the highly-virulent type I RHΔgra14 parasites in mice resulted in increased virulence. This study shows that GRA7, 14, and 15-induced host immunity via NFκB limits parasite expansion.

## Introduction

The obligate intracellular protozoan parasite *Toxoplasma gondii* can cause congenital toxoplasmosis, opportunistic infections in immunocompromised patients, and ocular disease (1–3). Epidemiological investigation of toxoplasmosis revealed that the majority of European and North American strains of the parasite belong to three distinct clonal lineages: type I, II, and III (4). These strains differ in virulence in mice: type I strains are the most virulent with a lethal dose (LD_100_) of one parasite, whereas the LD_50_ of type II and III strains are ~10^3^ and 10^5^, respectively (5). Previous studies demonstrated that virulence is largely mediated by several families of secretory pathogenesis determinants (6). These secreted effector proteins originate from different organelles, namely the rhoptries, known as rhoptry proteins (ROPs), and dense granules, known as dense granule proteins (GRAs) (7). Recently, it has become clear that *T. gondii* manipulates and modulates host resistance mechanisms at multiple points along pro-inflammatory pathways, which in turn dictates parasite burden and disease (8).

Nuclear factor-kappa B (NFκB), the central mediator of inflammatory responses and immune function, comprises homo- and heterodimers of five members: NFκB1 (p50), NFκB2 (p52), RelA (p65), RelB, and c-Rel (9, 10). The NFκB complex structure resides in the cytoplasm of unstimulated cells, where it is complexed with the inhibitor-κB (IκB) family of proteins, such as IκBα, IκBβ, and IκBε, which bind to the NFκB DNA binding domain and dimerization domain, the Rel homology domain, and thereby interfere with the function of the nuclear localization signal (11). Upon exposure to various infectious and inflammatory stimuli, the inhibitor proteins are phosphorylated, resulting in their ubiquitination and degradation, allowing the nuclear translocation of NFκB dimers to regulate gene expression (10). Many pathogens, including viruses, bacteria, and protozoa, have been reported to modulate the host NFκB pathway to optimize survival in the host (12).

Mice lacking c-Rel and RelB are highly susceptible to intraperitoneally infection with *T. gondii* and die within 10–15 days of infection, indicating the importance of the NFκB pathway for an adequate response to *T. gondii* infection (13, 14). C-Rel^−/−^ mice show an early defect in the number of IL-12p40-producing cells among the peritoneal exudates cells collected at 12, 24, and 48 h post-infection, although within 2–3 days this defect is no longer apparent (14). Moreover, increased susceptibility of c-Rel^−/−^ mice can be rescued by administration of IL-12 until 2 days post-infection, indicating that delayed production of IL-12 up to 2 days post-infection causes decreased production of IFN-γ and a failure to control the parasite burden (14). Despite these findings, modulation of the NFκB pathway by *T. gondii* remains to be further elucidated.

In this study, we used an NFκB-luciferase assay to screen candidates for their ability to regulate NFκB activity. We found that overexpression of GRA7, 14, and 15 in a type II strain resulted in strong NFκB activity; thus, we focused on these proteins. *Toxoplasma* GRA15 accounts for differences in NFκB activation among different strains (15). Recombinant GRA7 protein also has potent activity against the NFκB pathway; however, it is unclear whether endogenous GRA7 is capable of affecting the NFκB pathway (16). GRA14, which is secreted into the vacuole, can be transferred to both the parasitophorous vacuole (PV) membrane (PVM) and the intra-vehicular network (17). However, the molecular function of this protein remains unknown. Thus, the aim of this study was to gain a comprehensive understanding of the involvement of NFκB in the pathogenesis of toxoplasmosis by comparative analysis of three molecules that modulate inflammatory cytokines and chemokines, to ultimately aid the development of strategies to control chronic *Toxoplasma* infections.

## Materials and Methods

### Reagents

Anti-RelA (Sc-109) antibody was obtained from Santa Cruz Biotechnology (Santa Cruz, CA, USA). Anti-total IκBα (#9242), anti-phospho-IκBα (#2859), and anti- glyceraldehyde-3-phosphate dehydrogenase (GAPDH, #2118) were purchased from Cell Signaling Technology (Beverly, MA, USA).

### Ethics Statement

The use and care of animals complied with the Guide for the Care and Use of Laboratory Animals from the Ministry of Education, Culture, Sports, Science, and Technology, Japan. The experimental protocol was approved by the Committee on the Ethics of Animal Experiments at the Obihiro University of Agriculture and Veterinary Medicine (permit number: 19-50). All efforts were made to minimize animal suffering.

### Experimental Design

First, we constructed 17 GRAs and 21 ROPs expressing vectors. Then they were transiently transfected into 293T cells for monitoring NFκB activity. Next, 293T cells were infected with the parental Pru, PruΔ*gra7*, PruΔ*gra14*, and PruΔ*gra15* parasite strains and assessed their effect on NFκB activity. We evaluated the nuclear translocation of RelA in 293T cells overexpressing GRA7, GRA14, and GRA15 alone. Moreover, we also examined it in HFF cells infected with each parasite strain. Then, level of phosphorylated-IκBα in HFF cells infected with parasite strains were quantified. After that, we measured level of secreted IL-6 in Raw246.7 mouse macrophage cells infected with parasite strains, and then their RNA samples were supplied for transcriptome analysis. Lastly, we conducted survival test of both mice infected with type II *T. gondii* strains and mice infected with type I *T. gondii* strains.

### Parasites and Cell Culture

*Toxoplasma gondii* (type II, PruΔ*ku80*Δ*hxgprt* and type I, RHΔ*hxgprt*, RHΔ*hxgprt*Δ*gra7*, and RHΔ*hxgprt*Δ*gra14*) was maintained in monkey kidney adherent epithelial (Vero) cells in Eagle's minimum essential medium (MEM, Sigma, St. Louis, MO, USA) with 8% fetal bovine serum (FBS) and the appropriate antibiotics. RHΔ*hxgprt*Δ*gra7* and RHΔ*hxgprt*Δ*gra14* were kindly gifted by Prof. John Boothroyd (Stanford University School of Medicine) and Prof. Peter Bradley (University of California), respectively. Human embryonic kidney (293T) cells, human foreskin fibroblast (HFF) cells, and Raw264.7 mouse macrophages were cultured in Dulbecco's modified Eagle's medium (DMEM; Sigma) supplemented with 10% FBS and the appropriate antibiotics. For the purification of tachyzoites, infected cells were syringe-lysed using a 27-gauge needle to release the tachyzoite-stage parasites into the medium, which was then filtered using a 5.0-μm pore-sized filter (Millipore, Bedford, MA, USA).

### Plasmid Construction

All of the plasmids and primers used in this study are listed in Tables 1, 2. Further details of the plasmid construction can be found in the Supplemental Methods.

**Table 1:** Plasmids used in this study.

**Plasmid**	**Description**	**Use**	**Source or reference**
pBS/GFP/HX		Plasmid for cloning TgGRA7 knock-out vector	This study
pBS/GFP/TgGRA7KO/HX	HXGPRT cassette flaked by two homology arms from the 5'- and 3'- UTR of TgGRA7 gene	The knock-out vector targeting TgGRA7 gene	This study
pSAG1::CAS9-U6::sgUPRT	CAS9 expressed from the *Toxoplasma*SAG1 promoter and CRISPR gRNA targeting *Toxoplasma* UPRT produced from the U6 promoter	CRISPR plasmid targeting *Toxoplasma* UPRT	Addgene
pSAG1::CAS9-U6::sgTgGRA14	CAS9 expressed from the *TgSAG1* promoter and CRISPR gRNA targeting *TgGRA14* produced from the U6 promoter	CRISPR plasmid targeting between nucleotides 488 and 489 in *TgGRA14* gene	This study
pSAG1::CAS9-U6::sgTgGRA15	CAS9 expressed from the *TgSAG1* promoter and CRISPR gRNA targeting *TgGRA15* produced from the U6 promoter	CRISPR plasmid targeting between nucleotides 146 and 147 in *TgGRA14* gene	This study
pUPRT::DHFR-D	DHFR* cassette flanked by two homology arms from the 5′- and 3′-UTR of UPRT gene, respectively	Knockin the DHFR* expressed cassette into targeting gene	Addgene
p3XFLAG-CMV-14		Plasmid for cloning of FLAG tag fused gene	Sigma-Aldrich
p3XFLAG-CMV-*TgGRA1*	FLAG tag-fused *Tg(GOI)*	Luciferase reporter assay	This study
p3XFLAG-CMV-*TgGRA2*			This study
p3XFLAG-CMV-*TgGRA3*			This study
p3XFLAG-CMV-*TgGRA4*			This study
p3XFLAG-CMV-*TgGRA5*			This study
p3XFLAG-CMV-*TgGRA6*			This study
p3XFLAG-CMV-*TgGRA7*			This study
p3XFLAG-CMV-*TgGRA8*			This study
p3XFLAG-CMV-*TgGRA9*			This study
p3XFLAG-CMV-TgGRA11			This study
p3XFLAG-CMV-TgGRA12			This study
p3XFLAG-CMV-TgGRA14			This study
p3XFLAG-CMV-TgGRA15			This study
p3XFLAG-CMV-TgGRA16			This study
p3XFLAG-CMV-TgGRA23			This study
p3XFLAG-CMV-TgGRA24			This study
p3XFLAG-CMV-TgGRA25			This study
p3XFLAG-CMV-TgROP5			This study
p3XFLAG-CMV-TgROP8			This study
p3XFLAG-CMV-TgROP9			This study
p3XFLAG-CMV-TgROP10			This study
p3XFLAG-CMV-TgROP11			This study
p3XFLAG-CMV-TgROP12			This study
p3XFLAG-CMV-TgROP13			This study
p3XFLAG-CMV-TgROP14			This study
p3XFLAG-CMV-TgROP15			This study
p3XFLAG-CMV-TgROP16			This study
p3XFLAG-CMV-TgROP17			This study
p3XFLAG-CMV-TgROP18			This study
p3XFLAG-CMV-TgROP19A			This study
p3XFLAG-CMV-TgROP20			This study
p3XFLAG-CMV-TgROP23			This study
p3XFLAG-CMV-TgROP24			This study
p3XFLAG-CMV-TgROP26			This study
p3XFLAG-CMV-TgROP34			This study
p3XFLAG-CMV-TgROP35			This study
p3XFLAG-CMV-TgROP38			This study
p3XFLAG-CMV-TgROP39			This study
pGL4.32	Nuclear factor-κB response element (NF-κB)	Luciferase reporter assay for NF-κB signal	Promega
pGL4.74	Control Renillaluciferase expression vector		Promega
pBluescript SK (+)		Plasmid for cloning of *TgGRA14+UTR* gene	Add gene
pBluescript SK (+)-TgGRA7+UTR	*TgGRA14* expressed from the *TgGRA7* 5′UTR and 3′ UTR	Replacing the UPRT gene by *TgGRA7*	This study
pBluescript SK (+)-TgGRA14+UTR	*TgGRA14* expressed from the *TgGRA14* 5′UTR and 3′ UTR	Replacing the UPRT gene by *TgGRA14*	This study

**Table 2:** Primers used in this study.

**Primer**	**Sequence (5′-3′)**	**Use**
TgGRA1_cDNA_1F	ACC AGT CGA CTC TAG ATG GTG CGT GTG AGC GCT AT	To clone full length of the gene into XbaI and BamHI sites of the p3XFLAG-CMV-14 plasmid by In-Fusion cloning
TgGRA1_cDNA_2R	AGT CAG CCC GGG ATC TCT CTC TCT CTC CTG TTA AGA	
TgGRA2_cDNA_1F	ACC AGT CGA CTC TAG ATG TTC GCC GTA AAA CAT TG	
TgGRA2_cDNA_2R	AGT CAG CCC GGG ATC TCT GC GAA AAG TCT GGG ACG G	
TgGRA3_cDNA_1F	ACCA GTC GAC TCT AGA TGG ACC GTA CCA TAT GTC C	
TgGRA3_cDNA_2R	AGT CAG CCC GGG ATC TTT TCT TGG AGG CTT TGT CCA	
TgGRA4_cDNA_1F	ACC AGT CGA CTC TAG ATG CAG GGC ACT TGG TTT TC	
TgGRA4_cDNA_2R	AGT CAG CCC GGG ATC TCT CTT TGC GCA TTC TTT CCA	
TgGRA5_cDNA_1F	ACC AGT CGA CTC TAG ATG GCG TCT GTA AAA CGC GT	
TgGRA5_cDNA_2R	AGT CAG CCC GGG ATC TCT CTT CCT CGG CAA CTT CTT	
TgGRA6_cDNA_1F	ACC AGT CGA CTC TAG ATG GCA CAC GGT GGC ATC TA	
TgGRA6_cDNA_2R	AGT CAG CCC GGG ATC TAA AAT CAA ACT CAT TCA CAC	
TgGRA7_cDNA_1F	ACC AGT CGA CTC TAG ATG GCC CGA CAC GCA ATT TT	
TgGRA7_cDNA_2R	AGT CAG CCC GGG ATC TCT GGC GGG CAT CCT CCC CAT	
TgGRA8_cDNA_1F	ACC AGT CGA CTC TAG ATG GCT TTA CCA TTG CGT GT	
TgGRA8_cDNA_2R	AGT CAG CCC GGG ATC TAT TCT GCG TCG TTT GGA CGG	
TgGRA9_cDNA_1F	ACC AGT CGA CTCT AGA TGC GGT CAC TCA AGT CAA T	
TgGRA9_cDNA_2R	AGT CAG CCC GGG ATC TGA GTC CTC GGT CTT CCT GCG	
TgGRA11_212410_cDNA_1F	ACC AGT CGA CTC TAG ATG TCC CGC CGC ATG GCA TC	
TgGRA11_212410_cDNA_2R	AGT CAG CCC GGG ATC TTG GCT TCA ACT CGT CCT CTT	
TgGRA12_275850_cDNA_1F	ACC AGT CGA CTC TAG ATG GAG ACT GGC CTA AAG GA	
TgGRA12_275850_cDNA_2R	AGT CGC CCG GGA TCT CTT CTT TTG TGA AGG TTT C	
TgGRA14_cDNA_1F	ACC AGT CGA CTC TAG ATG CAG GCG ATA GCG CGG GG	
TgGRA14_cDNA_2R	AGT CAG CCC GGG ATC TTT CGC TTG GTC TCT GGT AGC	
TgGRA15_cDNA_1F	ACC AGT CGA CTC TAG ATG GTG ACA ACA ACC ACG CC	
TgGRA15_cDNA_2R	AGT CAG CCC GGG ATC TTG GAG TTA CCG CTG ATT GT	
TgGRA16_cDNA_1F	ACC AGT CGA CTC TAG ATG TAT CGA AAC CAC TCA GG	
TgGRA16_cDNA_2R	AGT CAG CCC GGG ATC TCA TCT GAT CAT TTT TCC GC	
TgGRA23_cDNA_1F	ACC AGT CGA CTC TAG ATG GCA GCG CGT GCG GGA AG	
TgGRA23_cDNA_2R	AGT CAG CCC GGG ATC TGT TCT TTC GCG CAA GGG GT	
TgGRA24_cDNA_1F	ACC AGT CGA CTC TAG ATG CTC CAG ATG GCA CGA TA	
TgGRA24_cDNA_2R	AGT CAG CCC GGG ATC TAT TAC CCT TAG TGG GTG GT	
TgGRA25_cDNA_1F	ACC AGT CGA CTC TAG ATG AAG CGT TTC TGG TTG TG	
TgGRA25_cDNA_2R	AGT CAG CCC GGG ATC TGT TTC TAT CGA ATT CCG GG	
TgROP5_cDNA_1F	ACC AGT CGA CTC TAG ATG GCG ACG AAG CTC GCT AG	
TgROP5_cDNA_2R	AGT CAG CCC GGG ATC TAG CGA CTG AGG GCG CAG CA	
TgROP8_cDNA_1F	ACC AGT CGA CTC TAG ATG TTT TCT GTG TTA CGT AA	
TgROP8_cDNA_2R	AGT CAG CCC GGG ATC TTG CCG GTT CTC CAT CAG TT	
TgROP9_cDNA_1F	ACC AGT CGA CTC TAG ATG ACG CAC CCA AAT CCC CT	
TgROP9_cDNA_2R	AGT CAG CCC GGG ATC TCT GCA TGA TCA ACG AGG GC	
TgROP10_cDNA_1F	ACC AGT CGA CTC TAG ATG GGA CGA CCC AGG TGG CC	
TgROP10_cDNA_2R	AGT CAG CCC GGG ATC TGT TGG GCG CAT CTT CCG TA	
TgROP11_cDNA_1F	ACC AGT CGA CTC TAG ATG TCG TCA TCC AGA TTG GT	
TgROP11_cDNA_2R	AGT CAG CCC GGG ATC TCC CCG TGA CGG GGA AGT AC	
TgROP12_cDNA_1F	ACC AGT CGA CTC TAG ATG GCA CGC GTT CTT CCT TG	
TgROP12_cDNA_2R	AGT CAG CCC GGG ATC TGA ACC GCC TCA AGA GAA AA	
TgROP13_cDNA_1F	ACC AGT CGA CTC TAG ATG AAG AGA ACA GAG CTT TG	
TgROP13_cDNA_2R	AGT CAG CCC GGG ATC TCA ATA GCC TCA AGG AAT TC	
TgROP14_cDNA_1F	ACC AGT CGA CTC TAG ATG TAT TCC TCC CCT CAG TC	
TgROP14_cDNA_2R	AGT CAG CCC GGG ATC TCA GCG CTT GCT TCT TCC TA	
TgROP15_cDNA_1F	ACC AGT CGA CTC TAG ATG CTG AAA ACG ACA CCT GC	
TgROP15_cDNA_2R	AGT CAG CCC GGG ATC TGA AAG GTG AGC TAT GAG GT	
TgROP16_cDNA_1F	ACC AGT CGA CTC TAG ATG AAA GTG ACC ACG AAA GG	
TgROP16_cDNA_2R	AGT CAG CCC GGG ATC TCA TCC GAT GTG AAG AAA GT	
TgROP17_cDNA_1F	ACC AGT CGA CTC TAG ATG GAG TTG GTG TTG TGC TT	
TgROP17_cDNA_2R	AGT CAG CCC GGG ATC TCT CCT TCT GTA ATA AAG CC	
TgROP18_cDNA_1F	ACC AGT CGA CTC TAG ATG TTT TCG GTA CAG CGG CC	
TgROP18_cDNA_2R	AGT CAG CCC GGG ATC TTT CTG TGT GGA GAT GTT CC	
TgROP19A_cDNA_1F	ACC AGT CGACTC TAG ATG AGA AGG CGC TGC TTT C	
TgROP19A_cDNA_2R	AGT CAG CCC GGG ATC TCT GAG ATC TGG ATG CGC GC	
TgROP20_cDNA_1F	ACC AGT CGA CTC TAG ATG CGC CTG GAT GCT GTG TA	
TgROP20_cDNA_2R	AGT CAG CCC GGG ATC TGT CAC TTG AAC TTG GCT CC	
TgROP23_cDNA_1F	ACC AGT CGA CTC TAG ATG GAA AAG ATC CTG TGG GC	
TgROP23_cDNA_2R	AGT CAG CCC GGG ATC TCT TGA TGC CTT TCA ACA GG	
TgROP24_cDNA_1F	ACC AGT CGA CTC TAG ATG GCA ACG CGT TCA TTC CT	
TgROP24_cDNA_2R	AGT CAG CCC GGG ATC TGG GAT TAC GGG AGA GTG TT	
TgROP26_cDNA_1F	ACC AGT CGA CTC TAG ATG TTG TTA AGC ATA TCT GC	
TgROP26_cDNA_2R	AGT CAG CCC GGG ATC TTA ATG GGG TAA ACA ACT GC	
TgROP34_cDNA_1F	ACC AGT CGA CTC TAG ATG ATG TTT CCT GCC GTC GC	
TgROP34_cDNA_2R	AGT CAG CCC GGG ATC TGC TCT CCT GTG CGT CTT CC	
TgROP35_cDNA_1F	ACC AGT CGA CTC TAG ATG CCG GAA CAA GAT CTT GC	
TgROP35_cDNA_2R	AGT CAG CCC GGG ATC TTT CGT TTT CCT GTT CAT GG	
TgROP38_cDNA_1F	ACC AGT CGA CTC TAG ATG AAA AAT ACT CTG TTG TC	
TgROP38_cDNA_2R	AGT CAG CCC GGG ATC TAA ATT GAT GCG TTC TTA TC	
TgROP39_cDNA_1F	ACC AGT CGA CTC TAG ATG AGC AAA CCT TTT TTC CC	
TgROP39_cDNA_2R	AGT CAG CCC GGG ATC TAA CAA TTG ACT CCC GAA GA	
TgROP41_cDNA_1F	ACC AGT CGA CTC TAG ATG CGT CAC GTG TTC AAC TC	
TgROP41_cDNA_2R	AGT CAG CCC GGG ATC TGG AAA GCA CTT GT GAG GTC	
TgGRA7_5UTR_1F	GTG GAT CCC ATG GAG ACA CAC GGT CAA CA	To clone 5′UTR of the TgGRA7 gene into pBS/GFP/HX
TgGRA7_5UTR_2R	CGA AGC TTT AAT GCA GCT GTC ATG TCT CG	
TgGRA7_3UTR_1F	ATGGGCCCGGTTGGAAAAGGACCCGTATG	To clone 3′UTR of the TgGRA8 gene into pBS/GFP/HX
TgGRA7_3UTR_2R	ATGGGCCCACGGAGACTGCCTTGTCTTTC	
TgGRA14II_484-gRNA	GAA GTT CTG AGC CGT TTC CTG TTT TAG AGC TAG AAA TAG C	Primer for CRISPR/CAS9 plasmids targeting the TgGRA14 gene (pSAG1::CAS9-U6::sgTgGRA14)
TgGRA15(II)_146-gRNA	GCT CGA TAA TTC GGT GGC TTG GGG TTT TAG AGC TAG AAA TAG C	Primer for CRISPR/CAS9 plasmids targeting the TgGRA15 gene (pSAG1::CAS9-U6::sgTgGRA15)
Common CAS9-U6-Rv	AAC TTG ACA TCC CCA TTT AC	Common primer for CRISPR/CAS9 plasmids targeting *Toxoplasma* genes
DHFR_GRA14_484_1F	AGG TTC AAG AAG TTC TGA GCC GTT TAA GCT TCG CCA GGC TGT AAA	To amplify an amplicon containing TgGRA14 homology regions surrounding a pyrimethamine-resistant DHFR* cassette
DHFR_GRA14_484_2R	CAG ACG CAA CAG AAC CAA GGG GAA TTC ATC CTG CAA GTG CAT AG	
DHFR-25ntTgGRA15(II)_146_1F	CAA GTC ACG CTC GAT AAT TCG GTG GAA GCT TCG CCA GGC TGT AAA	To amplify an amplicon containing TgGRA15 homology regions surrounding a pyrimethamine-resistant DHFR* cassette
DHFR-21ntTgGRA15(II)_146_2R	GAG CAC CGT AAG ATA CCC AAG GGA ATT CAT CCT GCA AGT GCA TAG	
TgGRA7-KOS-1F	CGT CAT GAG TAC CGG GAC AT	To confirm the correct homologous recombination of HXGPRT cassette with the TgGRA7 gene
TgGRA7-KOS-2R	ATT CAG ACC TGC TGC GAG CC	
TgGRA7-KOS-3R	GCA AGG AAC GAT CAT GCG TG	
HXGPRT-KOS-1F	CTTGTCGGGGAGCAACAGCC	
TgGRA7_RT_1F	TCA CCA CCA GCA TGG ATA AGG	To confirm the insertion of TgGRA7+UTR cassette into the TgUPRT gene
TgGRA7_RT_2R	GCC TCG CTT CCT GAA ATG AAC	
TgGRA7+UTR_467-2R	GAT TTT CAG CCA CGC CTG TC	To confirm the insertion of TgGRA7+UTR cassette into the TgUPRT gene
TgGRA7+UTR_467-1F	AAG GAC CCG TAT GCA GGT AGC T	
TgGRA14screen-1Fv3	CGA GTT GTA GCT GG CTT TTC	To confirm the insertion of DHFR* cassette into the TgGRA14 gene
TgGRA14screen-1Rv3	TGT CAC GGG GAG ACT AGC GT	
TgGRA15(II)_screen_1F	TTT CCA GGA GGA ATC GCG CC	To confirm the insertion of DHFR* cassette into the TgGRA15 gene
TgGRA15 (II)_screen_2R	CTG CCT CGT CGT GTT TCC CG	
DHFR2-1F	CCA TTG TGA ACA TCC TCA AC	To confirm the insertion of DHFR* cassette into the target gene
TgDHFR-TS_screen_2R	CAG ACA CAC CGG TTT CTG CAT	
TgGRA7(II)+UTR_1F	ATG CGG CCG CAG GAA AAC AGT GTT TCC GAA	To clone full length of the TgGRA7 containing UTR region into Not1 and EcoR5 sites of the pBluescript+SK plasmid by Iigation cloning (pBluescript-TgGRA7+UTR)
TgGRA7(II)+UTR_2R	ACG ATA TCA TGC GTC TTT TGT AGT GAA T	
TgGRA14(II)+UTR_1Fv2	ATT CTA GAA AAT AAT GTG CGC ACA CAA C	To clone full length of the TgGRA14 containing UTR region into Not1 and EcoR5 sites of the pBluescript+SK plasmid by Iigation cloning (pBluescript-TgGRA14+UTR)
TgGRA14(II)+UTR_2Rv2	TCA TCG ATT GCC AGC TCC TTT CAG CTT C	
pBlue_UPRT_1F	TGT GGC GTC TCG ATT GTG AGA TAG GGC GAA TTG GAG CTC C	To amplify an amplicon containing TgUPRT homology region surrounding TgGRA14+UTR expressed cassette
pBlue_UPRT_2R	TTT CCA TCG ACT CGC CAG CTA GGG AAC AAA AGC TGG GTA C	
UpgRNA-1F	GAT CCG CTT CTC TTG TAC TGC	To confirm the insertion of TgGRA14+UTR cassette into the TgUPRT gee
DngRNA-2R	AAG CAG GTG CAG CGG ACA AG	
CXCL1_RT_1F	CAA TGA GCT GCG CTG TCA GT	Real-time PCR for expression of mouse CXCL1 mRNA
CXCL1_RT_2R	TTG AGG TGA ATC CCA GCC AT	
CXCL5_RT_1F	CGC TAA TTT GGA GGT GAT CCC	Real-time PCR for expression of mouse CXCL5 mRNA
CXCL5_RT_2R	ACT TCC ACC GTA GGG CAC TG	
IL1-beta-RT-F1	CCA AAA GATGAA GGG CTG CT	Real-time PCR for expression of mouse IL-1beta mRNA
IL1-beta-RT-R1	TCA TCT GGA CAG CCC AGG TC	
IL6-RT-F1	TTC CAT CCA GTT GCC TTC TTG	Real-time PCR for expression of mouse IL-6 mRNA
IL6-RT-R2	GAA GGC CGT GGT TGT CAC C	
CCL17_RT_1F	ATG TAG GCC GAG AGT GCT GC	Real-time PCR for expression of mouse Ccl17 mRNA
CCL17_RT_2R	TGA TAG GA ATG GCC CCT TTG	
CCL7_RT_1F	GGA TCT CTG CCA CGC TTC TG	Real-time PCR for expression of mouse CCL7 mRNA
CCL7_RT_2R	GGC CCA CAC TTG GAT GCT	
LCN2-RT-F1	CCA GTT CGC CAT GGT ATT TTT C	Real-time PCR for expression of mouse LCN2beta mRA
LCN2-RT-R1	CAC ACT CAC CAC CCA TTC AGT T	
CSF3-RT-F1	CTG GCA GCA GAT GGA AAA CC	Real-time PCR for expression of mouse CSF3 mRNA
CSF3-RT-R2	TGT GTG GGC TGC ACA GTA GG	
Ptgs2_RT_1F	ATG TAG GCC GAG AGT GCT GC	Real-time PCR for expression of mouse PTGS2 mRNA
Ptgs2_RT_2R	CCA GCA CTT CAC CCA TCA GTT	
GAPDH-RT-1F	CCC AGG TCC TCG CTT ATG ATC	Internal control gene for real-time RT-PCR analysis
GAPDH-RT-2R	CCT GCT TCA CCA CC TTC TTG AT	

### Luciferase Assay in 293T Cells Expressing *Toxoplasma* Genes

293T cells in a 96-well plate were transfected with pGL4.32[luc2P/NF-κB-RE/Hygro] (Promega, Madison, WI, USA), together with the pGL4.74[hRluc/TK] vectors (Promega) and the mammalian expression plasmids of each parasite molecule, respectively, using Fugene HD (Promega). The empty p3 × FLAG-cmv14 vector was used as a negative (empty) control. At 18 h post-transfection, the luciferase activities of the total cell lysates were measured with the Dual-Glo luciferase assay system (Promega).

### Generation of PruΔ*gra7* PruΔ*gra14*, and PruΔ*gra15* Deletion Mutants, and GRA7- and GRA14-Complemented Strains

The knock-out plasmid (pBS/GFP/TgGRA7KO/HX) was transfected into parental Pru strains, and selected with 25 μg/ml 3-mercaptopropionlc acid and 50 μg/ml xanthine. The electroporation of tachyzoites was performed as described previously (18). The drug-resistant parasites were cloned by limiting dilution and tested by PCR (Supplemental Figure 1). PCR-positive clones were further analyzed with western blotting and indirect fluorescent antibody test (IFAT) to confirm the protein expression. To disrupt GRA14 and GRA15 in Pru, we cotransfected the parasite with 50 μg of the CRISPR plasmid (pSAG1::CAS9-U6::sgTgGRA14 and pSAG1::CAS9-U6::sgTgGRA15), along with an amplicon containing homologous regions of GRA14 and GRA15 surrounding a pyrimethamine-resistant dihydrofolate reductase (DHFR^*^) cassette (5 μg), respectively. Insert fragments were prepared by PCR amplification using the primers listed in Table 2. Selection by growth for 10 to 14 days in pyrimethamine (1 μM) was used to obtain stably resistant parasite clones that were subsequently screened by PCR to ensure the correct integration of DHFR^*^ into the GRA14 and GRA15 gene loci (Supplemental Figure 1). PCR-positive clones were further analyzed by western blotting and IFAT to confirm the loss of GRA14 expression (Supplemental Figure 3). To complement the GRA7 and GRA14 genes, we transfected GRA7- and GRA14-deficient parasites with pSAG1::CAS9-U6::sgUPRT (50 μg) to target integration to the UPRT locus, along with an amplicon containing the TgGRA7 and TgGRA14 genes containing the 5′- and 3′-untranslated regions (UTRs) (5 μg), respectively. Stably resistant clones were selected by growth on fluorouracil (10 μM) for 10 to 14 days and were subsequently screened by PCR to ensure the correct integration into the UPRT gene locus (Supplemental Figure 1). PCR-positive clones were further analyzed by western blotting and IFAT to confirm the protein expression (Supplemental Figure 2).

### Cytokine ELISA

Raw246.7 mouse macrophage cells in a 12-well plate were infected with parasite lines (multiplicity of infection = 0.5) for 24 h, along with control uninfected cells. Then, supernatants were collected and IL-6 levels were determined using a cytokine enzyme-linked immunosorbent assay (ELISA) kit (Mouse OptEIA ELISA set; BD Biosciences, San Jose, CA, USA).

### RNA Sequencing and KEGG Pathway Enrichment Analysis

Raw246.7 mouse macrophage cells were infected with parasite lines for 24 h, then cells were lysed and total RNA was extracted using TRI reagent (Sigma). Library preparation was performed using a TruSeq stranded mRNA sample prep kit (Illumina, San Diego, CA, USA). Sequencing was performed on an Illumina HiSeq 2500 platform in a 75-base single-end mode. Illumina Casava1.8.2 software was used for base calling and raw sequence reads were subjected to quality control, then the cleaned reads were mapped to the reference mouse genome (mm10) with CLC Genomics Workbench version 10 (GWB; CLC bio, Aarhus, Denmark) (read mapping parameters: minimum fraction length of read overlap = 0.95 and minimum sequence similarity = 0.95). Only uniquely mapped reads were retained for further analysis. We identified differentially expressed genes (DEGs) as described in detail previously (19). The expression of each gene was compared among parasite lines using the differential expression for RNA-seq function in CLC GWB. DEGs were identified as genes with a fold change in expression of >2, and a max group mean of >1. KEGG pathway analysis was also conducted as described in detail in a previous article (19). The list of DEGs was subjected to a KEGG pathway enrichment analysis using the clusterProfiler package (20) in the statistical environment R to assess their overarching function. Following CPM normalization, the expression of each gene in the enriched pathways was normalized with Z-score normalization and visualized. Normalized gene expression was visualized in a heatmap using the heatmap.2 function (21) in the gplots package in R. The genes were hierarchically clustered based on the Pearson correlation distance and the group average method.

### IFAT in *T. gondii*-Infected Cells

HFF cells in a 12-well plate were infected with parasites (multiplicity of infection = 1) for 24 h, along with uninfected control cells. The cells were then fixed with 4% (vol/vol) paraformaldehyde in PBS for 15 min at room temperature, permeabilized with 0.1% (vol/vol) Triton X-100 and blocked in PBS with 3% (wt/vol) bovine serum albumin. Cover slips were incubated with primary antibody for 1 h at room temperature, and fluorescent secondary antibody for 1 h at room temperature. Nuclei were counterstained with Hoechst dye. Coverslips were then mounted onto the glass slide with Mowiol 4-88 (Sigma), and photographs were taken using All-in-One microscopy (BZ-9000, Keyence, Itasca, IL, USA). Quantification of the nuclear signal was performed by randomly selecting at least 20 infected cells per *T. gondii* strain and measuring the mean signal intensity per nucleus using the BZ analyzer II (Keyence).

### IFAT in 293T Cells With Forced Expression of GRA Proteins

The 293T cells in a collagen 1-coated 12-well plate were transiently transfected with expression vectors of GRA7, GRA14, or GRA15, or the empty p3 × FLAG-cmv14 vector as a negative (empty) control, using Fugene HD. After 24 h, IFAT and quantification of the nuclear signal were performed as described above.

### Western Blotting

HFF cells were infected with parasites (multiplicity of infection = 3) for 24 h, then lysed using the LysoPure™ Nuclear and Cytoplasmic Extractor Kit (Wako, Osaka, Japan) supplemented with complete mini protease inhibitors and Phos stop (Roche, Mannheim, Germany). The cell lysates were separated by SDS-polyacrylamide gel electrophoresis and transferred to a Poly Vinylidene Di-Fluoride membrane (Millipore), which was blocked in TBS/0.1% Tween-20/2% ECL Prime Blocking Reagent (GE Healthcare, Buckinghamshire, UK) and incubated with primary and secondary antibodies. The protein bands were visualized by ECL Prime Western Blotting Detection reagent (GE Healthcare), and analyzed by Versa Doc with Quantity One (Bio-Rad, Munich, Germany). Band intensity was quantified using ImageJ software developed by the US National Institutes of Health.

### Survival of Mice Infected With *Toxoplasma gondii*

Male C57BL/6J mice, of 8 weeks of age, were obtained from Clea Japan (Tokyo, Japan). Mice were infected intraperitoneally with 500 tachyzoites of the parental strain Pru, or mutant strains PruΔ*gra7*, PruΔ*gra14*, or PruΔ*gra15*. Mice were also infected intraperitoneally with 10,000 parental Pru or PruΔ*gra14* parasites. To determine the survival rates to the type I RH strain, 500 tachyzoites the RHΔ*gra14*, RHΔ*gra7*, or their parental parasites were injected into the right footpads of mice and their survival was monitored for up to 30 days.

### Statistical Analyses

Statistical analyses were performed using GraphPad Prism (version 6.0) software (GraphPad Software, San Diego, CA, USA). Statistically significant differences among groups were determined using one-way ANOVA with Tukey's *post-hoc* test. *P*-values of < 0.05 represent statistically significant differences. The survival rate was compared between groups using the log-rank test.

## Results

### Ectopic Expression of Type II GRA14 Activates NFκB Signaling in 293T Cells

**Figure 1:**
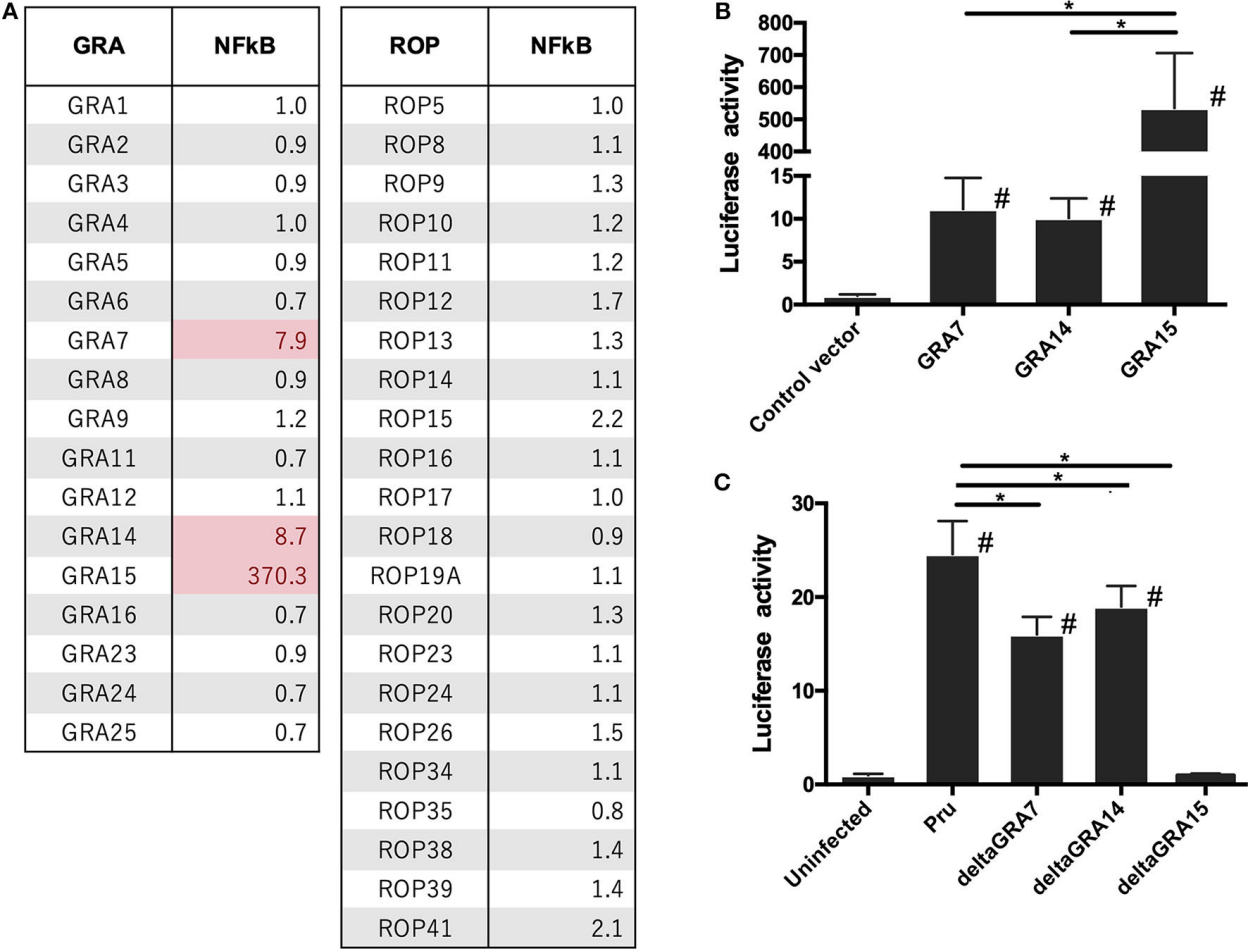
Luciferase activities in 293T cells transfected with NFκB reporter plasmid. 293T cells were transiently transfected with pGL4.32 expressing firefly luciferase and pGL4.74 expressing renilla luciferase. **(A,B)** Cells were immediately transfected with the expression vectors of GRAs and ROPs, and the empty p3×FLAG-cmv14 vector used as a negative (empty) control. The promoter activity was determined and is shown as a fold-increase in the luciferase activity normalized for Renilla luciferase activity. **(C)** Pru, PruΔ*gra7* (deltaGRA7), PruΔ*gra14* (deltaGRA14), or PruΔ*gra15* (deltaGRA15) lines were added to the cells. After 12 h, parasites were added to the host cells, lysates were prepared, and luciferase activity was measured. The promoter activity was determined and is shown as a fold-increase in the luciferase activity normalized for Renilla luciferase activity. Values are the means ± SD of triplicate samples, *p < 0.05. #a significant difference with the control vector and or uninfected cells (*p* < 0.05). Differences were tested by one-way ANOVA with turkey's *post-hoc* test in **(B,C)**. Data are representative of two independent experiments.

To investigate which molecules modulate the NFκB pathway in *Toxoplasma*, we constructed mammalian expression vectors for 17 GRAs and 21 ROPs of a *Toxoplasma* type II strain. Then, we assessed whether their overexpression, together with luciferase reporter plasmids carrying an element dependent on the NFκB promoter, activated the reporter. Overexpression of GRA7, GRA14, and GRA15 activated NFκB (Figure 1A). Overexpression of GRA14 stimulated the NFκB promoter to a similar level as that of GRA7, whereas GRA15 produced much higher levels of NFκB-dependent luciferase activity than GRA7 and GRA14 (Figure 1B). The expression of these molecules in 293T cells was confirmed by western blotting (Supplemental Figure 3). Thus, we focused on GRA7, GRA14, and GRA15 for further analysis.

Next, we generated PruΔ*gra7*, PruΔ*gra14*, and PruΔ*gra15* parasites based on the gene-editing strategies depicted in Supplemental Figure 2. We isolated single clones of drug-resistant parasites and performed diagnostic PCR to check for correct integration (Supplemental Figure 1). Moreover, we established complementation of GRA7 and GRA14. The GRA7 and GRA14 expression cassettes containing the 5′ UTR and 3'UTR were inserted into the UPRT gene locus. Drug-resistant clones were isolated and correct integration into the UPRT locus was confirmed (Supplemental Figure 1). Clones, with the exception of PruΔ*gra15*, were further analyzed by an IFAT and western blotting to confirm the protein expression (Supplemental Figure 2). The PruΔ*gra15* mutant was excluded because of the lack of an anti-GRA15 antibody. We then assessed the physiological changes in the transgenic lines *in vitro*. The infection rates and egress rates of the PruΔ*gra7* and PruΔ*gra14* strains in Vero cells were similar to those of the parental strain (Supplemental Figures 5A–D). Whereas, the *in vitro* replication rate of PruΔ*gra7* parasites was significantly higher than that of the parental parasites (Supplemental Figure 5E). The replication rate of PruΔ*gra14* was comparable to that of the parental parasites (Supplemental Figure 5F).

Next, we analyzed how each GRA contributes to NFκB activation because it is known that GRA15 plays a dominant role in NFκB activation by type II *T. gondii*. Cells infected with the PruΔ*gra7* and PruΔ*gra14* mutants showed a partial decrease in luciferase activity compared with cells infected with the parental Pru (Figure 1C). However, for PruΔ*gra15*, NFκB activity was abolished in the infected cells (Figure 1C).

### Each GRA Expression Alone Is Sufficient to Activate NFκB in 293T Cells

**Figure 2:**
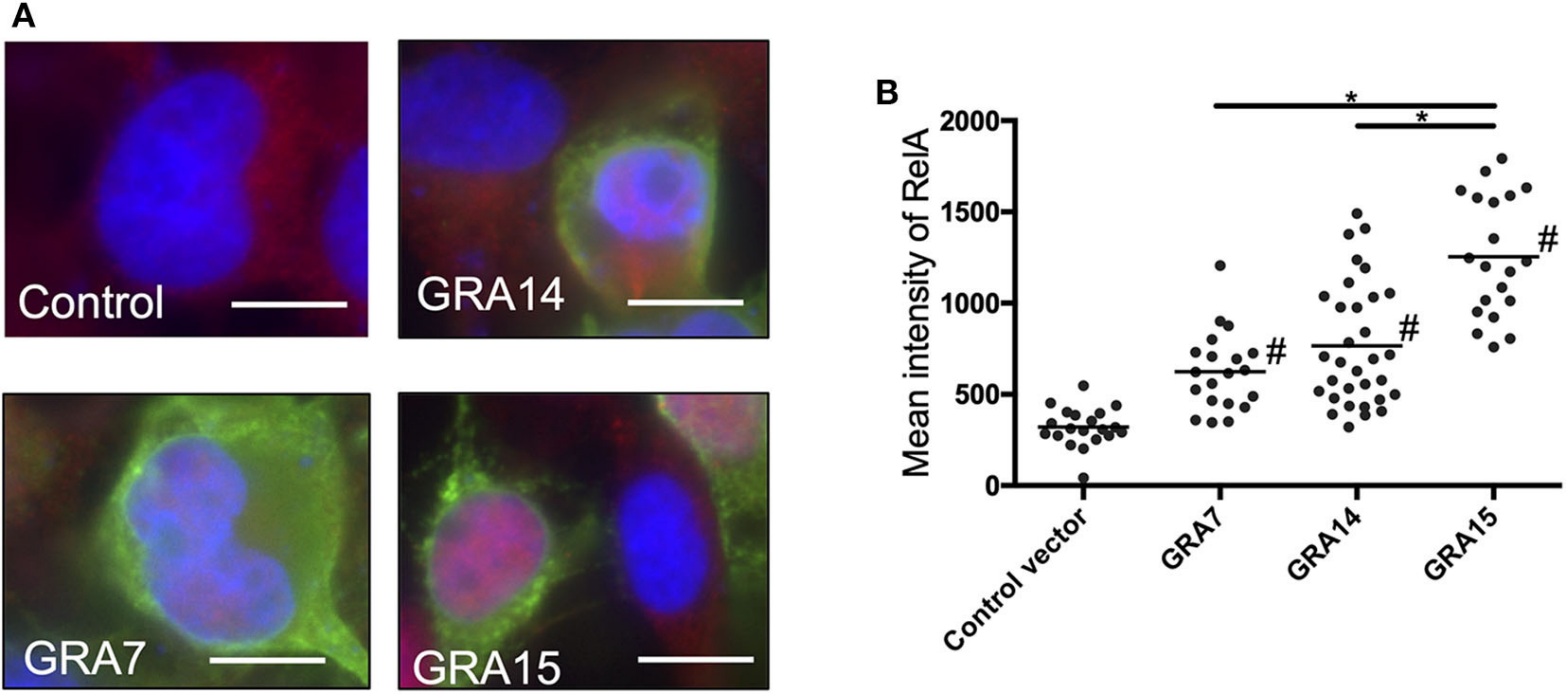
GRA expression activates nuclear translocation of NF-κB RelA in 293T cells. **(A)** 293T cells were transfected with the expression vectors for GRA7, GRA14, or GRA15, or the empty p3 × FLAG-cmv14 vector used as a negative (empty) control. Cells were then fixed and stained with α-NFκB RelA (red), α-FLAG (green), or Hoechst dye (blue). Bars, 10 μm. **(B)** The mean intensity of RelA in the nucleus was measured for at least 20 cells per group. Bar indicates the mean of each group, *p < 0.05. #a significantly higher level of nuclear RelA compared with the control cells (*p* < 0.05). Differences were tested by one-way ANOVA with turkey's *post-hoc* test. Experiments were performed twice.

We assessed whether each GRA protein alone is sufficient to activate the process of NFκB signal transduction. The level of nuclear RelA in GRA7- or GRA14-expressing cells was significantly higher than the level in control cells (Figure 2). Moreover, the level of RelA nuclear translocation in cells expressed GRA15 was even higher than that in cells expressing GRA7 and GRA14 (Figure 2). First, we performed transient expression of each GRA gene. However, it is uncertain whether the function of ectopic single parasite molecule is the same as that of its native molecule. In addition, western blotting in the Supplemental Figure 3 indicated different expression levels among the transfection with GRA genes. Thus, we conducted similar experiments using deficient parasite strains.

**Figure 3:**
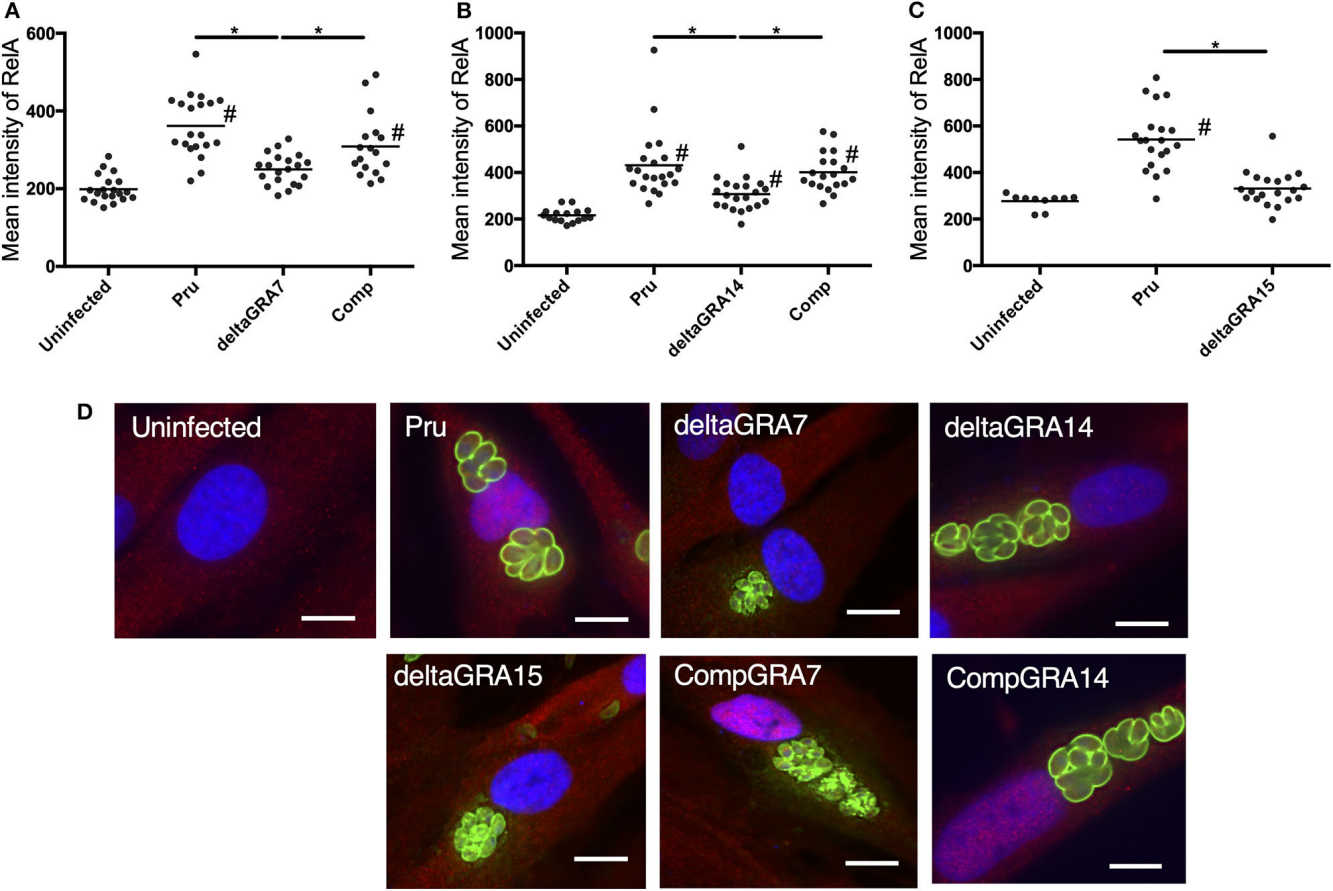
*T. gondii* infection with parasites deficient in GRA7, 14, or 15 decreases nuclear translocation of NFκB RelA in HFF cells. **(A–C)** The mean intensity of RelA in the nucleus was measured in HFF cells for each group. Experiments were performed twice. Bar indicates the mean of each group, *p < 0.05. #a significantly higher level of nuclear RelA compared with uninfected cells (*p* < 0.05). Differences were tested by one-way ANOVA with turkey's *post-hoc* test. **(D)** Representative IFAT images of HFF cells infected with parasite strains and uninfected control cells. After infection for 24 h, cells were fixed and stained with α-NFκB RelA (red), α-SAG1 (green), and Hoechst dye (blue). Bars, 10 μm.

**Figure 4:**
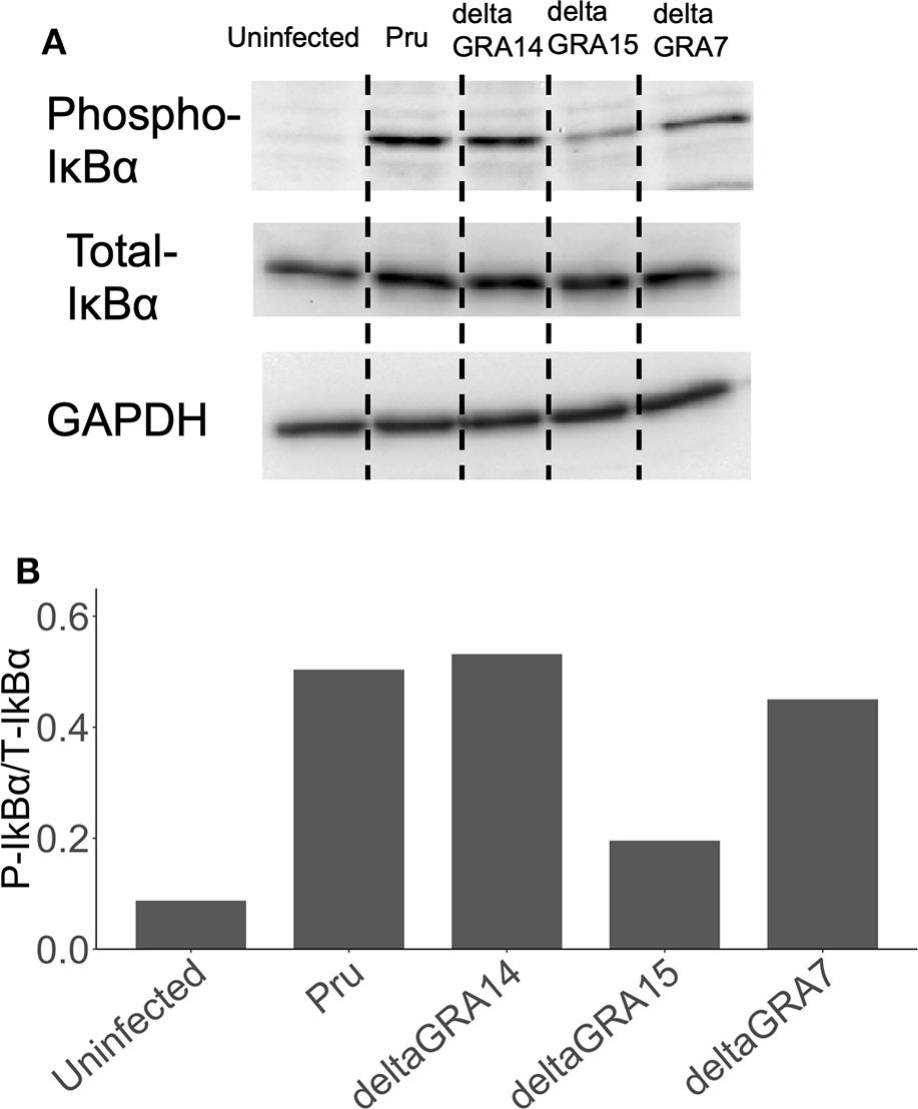
Levels of phosphorylated IκBα in HFF cells infected with *T. gondii* strains. **(A)** HFF cells were infected with parasite strains for 24 h, then cell lysates were collected, separated on an SDS-PAGE gel, and western blot analysis was carried out with anti-phospho-IκBα, total IκBα, and GAPDH (host cell loading control) antibodies. **(B)** The ratio of phospho-IκBα/total IκBα in cells stimulated with parasites and uninfected cells. This experiment was repeated twice with similar results.

Cells infected with the parental Pru strain revealed a higher level of nuclear RelA than cells infected with PruΔ*gra7* or PruΔ*gra14*, whereas the complemented strain showed a similar level of RelA signal to the parental parasite (Figures 3A,B). Moreover, GRA15 deletion almost abolished the nuclear translocation of RelA (Figure 3C). Representative images from these experiments are shown in Figure 3D. Next, we assessed whether each GRA affects the phosphorylation of IκBα by western blotting. The phosphorylated IκBα levels were comparable between cells infected with the parental, PruΔ*gra14* and PruΔ*gra7* strains, whereas GRA15 deficiency obviously reduced phosphorylated IκB (Figure 4). The relative levels of phosphorylated IκBα compared with the parental Pru-infected cells were 89, 105, and 38% in cells infected with PruΔ*gra7*, PruΔ*gra14*, and PruΔ*gra15* strains, respectively (Figure 4).

### Deficiency of GRA7, GRA14, and GRA15 Predominantly Results in Downregulation of Gene Expression Mediated by NFκB in Macrophages Infected With *T. gondii*

**Figure 5:**
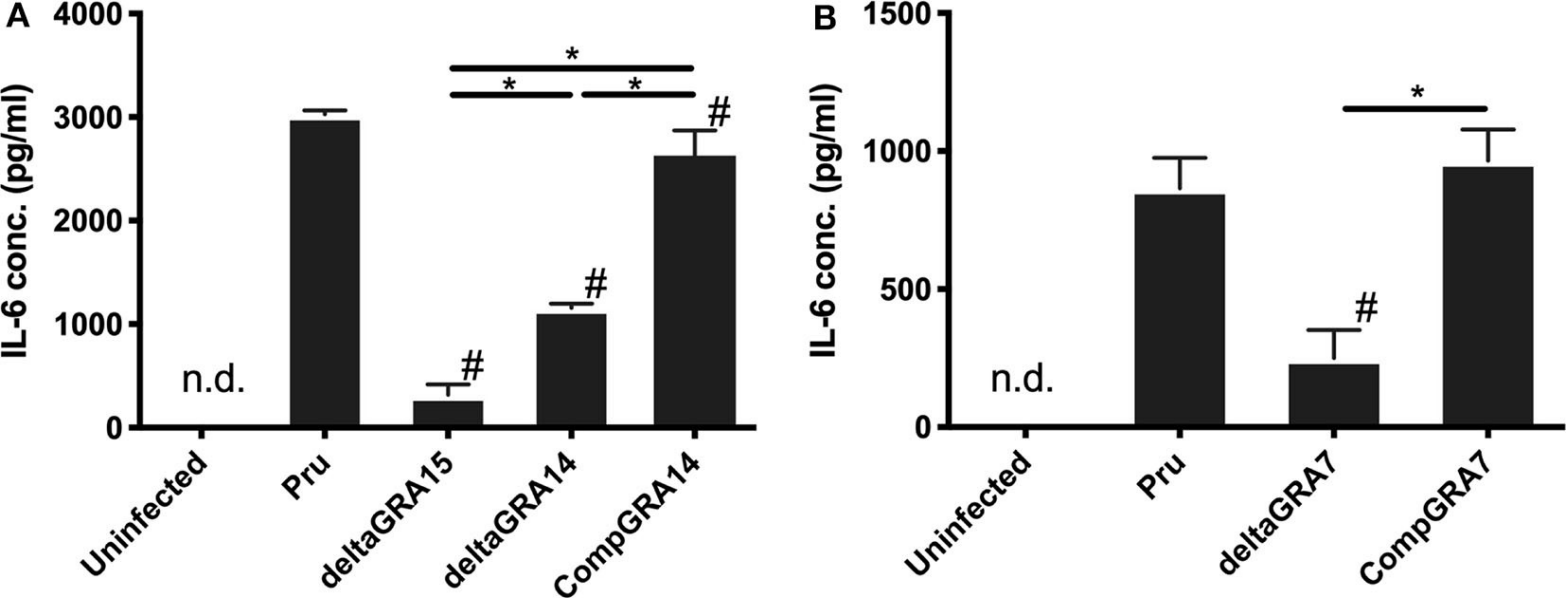
Levels of interleukin-6 in Raw246.7 macrophage cells. **(A)** Raw246.7 macrophage cells were infected with the parental Pru, PruΔ*gra15*, PruΔ*gra14*, and GRA14 complemented parasite strains. **(B)** Raw246.7 macrophage cells were infected with the parental Pru, PruΔ*gra7*, and GRA7 complemented parasite strains. **(A,B)** At 24 h post-infection, supernatants were collected and IL-6 levels were determined by cytokine ELISA. These experiments were performed three times using triplicate samples. Values are the means ± SD of triplicate samples, * indicates a significant difference (*p < 0.05). #a significantly lower level of IL-6 compared with the Pru strain infected cells (*p* < 0.05). Differences were tested by one-way ANOVA with turkey's *post-hoc* test. Experiments for PruΔ*gra14* and the complemented lines were performed in tandem with PruΔ*gra15*, and experiments for PruΔ*gra7* and the complemented lines were performed independently.

**Figure 6:**
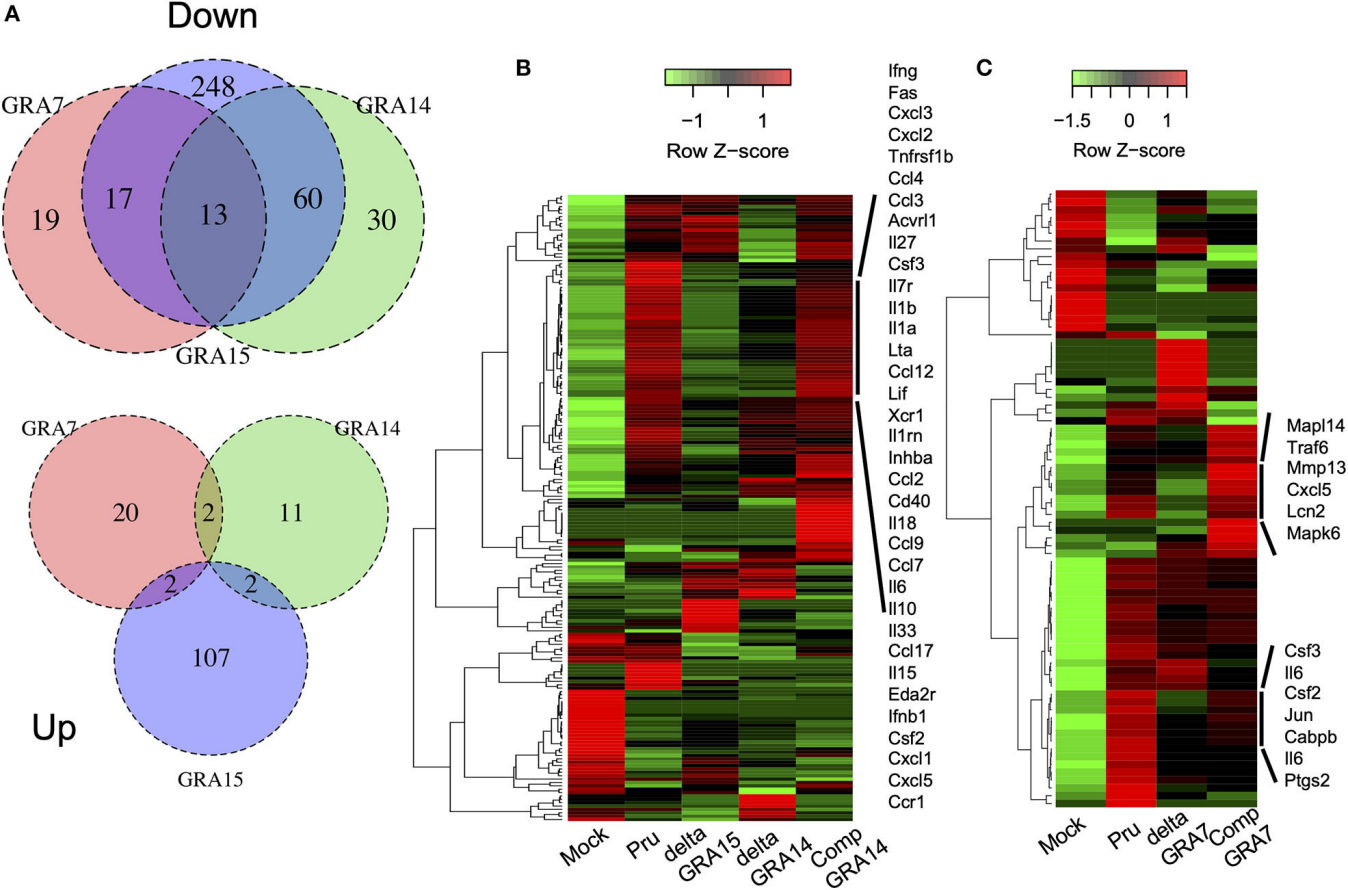
GRA deficiency resulted in downregulation of gene expression mediated by NFκB. RNA-seq analysis of Raw246.7 macrophage cells infected for 24 h with parasite strains and the uninfected cells (Mock) (*n* = 1 per group). DESeq analysis identified genes with a more than 2-fold change in expression between the GRA deficient strain and the parental Pru and complemented strains following infection. **(A)** Venn diagrams comparing GRA7, GRA14, and GRA15-dependent DEGs during *T. gondii* infection: down, downregulated; up, upregulated. **(B)** Heatmap showing that the most significantly enriched pathway associated with the GRA14 expression status was the cytokine-cytokine receptor interaction, which contained a subset of 41 genes. Rows represent samples from different processes, and columns represent genes. **(C)** Heatmap showing that the most significantly enriched pathway associated with the GRA7 expression status was the IL17 signaling pathway, which contained a subset of 91 genes. Rows represent samples from different processes, and columns represent genes. The cluster shown in **(B,C)**, which was upregulated when GRA14 or GRA7 expression was restored, is enlarged on the right. The experiment for GRA14 was performed in tandem with GRA15, and the experiment for GRA7 was performed independently.

We next analyzed the levels of interleukin-6 (IL-6) in mouse macrophage Raw246.7 cells infected with parasites, and found that not only GRA15 deficiency but also GRA7 and GRA14 deficiency decreased the level of secreted IL-6 in the culture supernatant (Figure 5). This result indicated that all of these GRAs affect the induction of the host immune response. To determine the host gene expression profiles relevant to these GRAs, we conducted transcriptome analysis of Raw246.7 cells infected with each strain and the uninfected cells. In total, 49, 103, and 338 genes were downregulated and 24, 15, and 111 genes were upregulated by GRA7, GRA14, and GRA15 deficiency, respectively (Figure 6A, the complete sets of genes are listed in Supplemental Data Sheet 1). A Venn diagram was created to illustrate the similarities and differences among the genes regulated by these three GRAs (Figure 6A). This indicated that a number of common genes were regulated by these GRAs, and that GRA15 deficiency had more diverse effects than GRA7 and GRA14 deficiency.

To gain greater insight into the pathways regulated by each GRA in host cells, we conducted Kyoto Encyclopedia of Genes and Genomes (KEGG) pathway analysis on the relevant genes. This analysis primarily identified immune-response-related pathways, such as the cytokine-cytokine receptor interaction pathway, the IL-17 signaling pathway, and the tumor necrosis factor (TNF) signaling pathway, that were significantly enriched in the DEGs downregulated in macrophage cultures infected with deficient parasites compared with their expression in cell cultures infected by Pru and the complemented parasites (Supplemental Data Sheet 2). A heatmap of the gene expression associated with the cytokine-cytokine receptor interactions illustrated that GRA14, similar to GRA15, regulated some cytokines and chemokines (Figure 6B). In addition, a heatmap of the gene expression associated with the IL-17 signaling pathway defined several cytokines and chemokines as GRA7-regulated genes (Figure 6C, the complete sets of genes are available in Supplemental Data Sheet 3. To confirm the host genes whose expression is regulated by GRAs, we quantified the expression levels of several genes: IL-1β, IL-6, Cxcl1, Cxcl5, and Ccl17 for GRA14 and GRA15; and IL-6, Ccl7, Lcn2, Csf3, and Ptgs2 for GRA7. These genes were selected because their expression appeared to be regulated by GRAs according to the heatmaps (Supplemental Data Sheet 3). In most cases, the gene expression profiles were consistent between the transcriptome and the real-time PCR data (Supplemental Figure 6). Collectively, these results indicated that GRA7, GRA14, and GRA15 deficiency robustly downregulated the immune response-related pathways induced by *T. gondii* infection.

### GRA7, GRA14, and GRA15 Deficiency Increased Parasite Virulence in Mice

**Figure 7:**
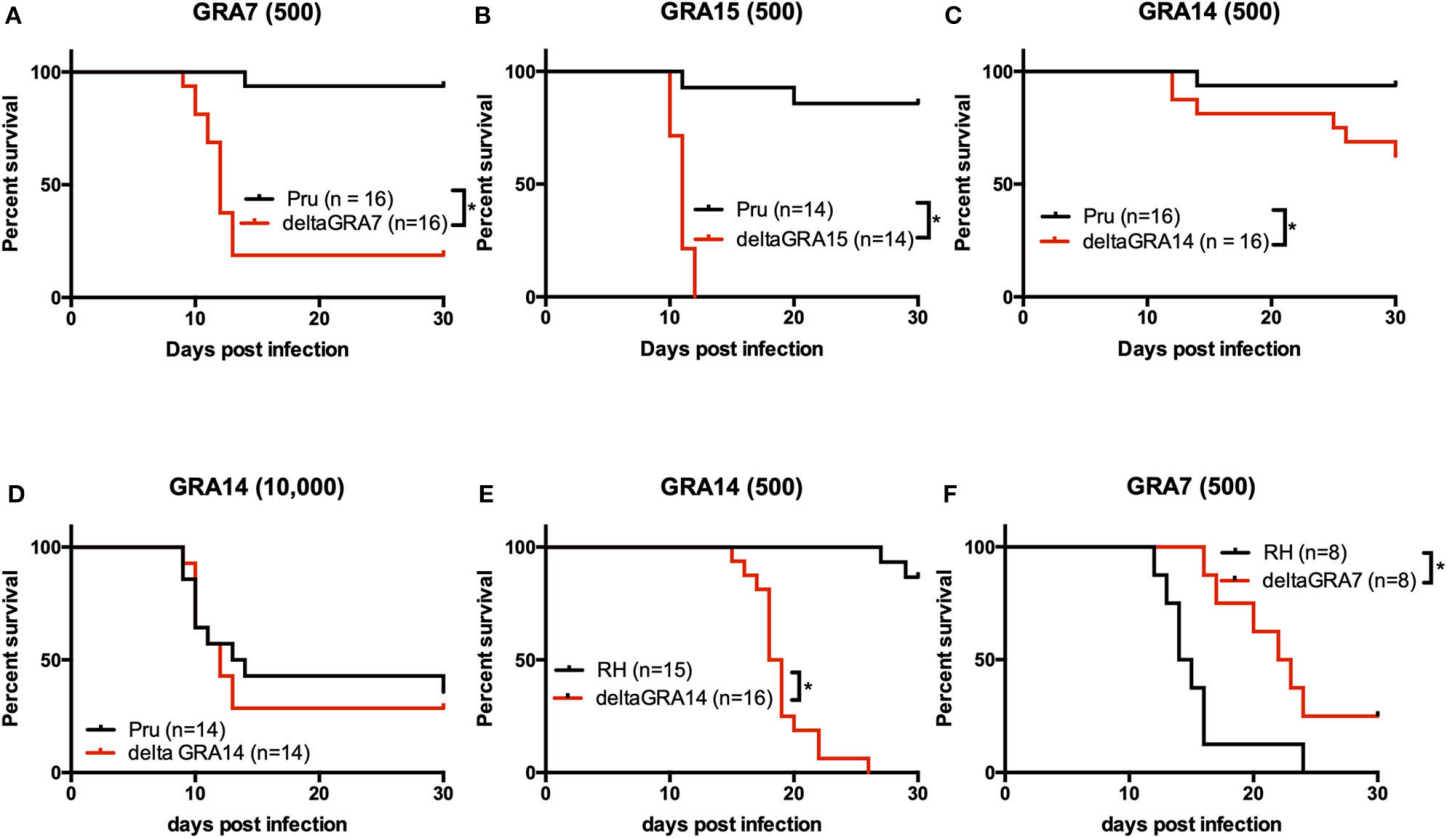
Survival in mice infected with *T. gondii*. **(A–E)** Mice were intraperitoneally infected with a low dose (500) or a high dose (10^4^) of *T. gondii* tachyzoites of parasites strains, and survival was monitored for 30 days. **(A)** Survival rate of mice infected with 500 tachyzoites. In total, 16 mice were infected per strain (6 + 10). Data are summarized from two independent experiments. **(B)** Survival rate of mice infected with 500 tachyzoites. In total, 14 mice were infected per strain (6 + 8). Data are summarized from two independent experiments. **(C)** Survival rate of mice infected with 500 tachyzoites. In total, 16 mice were infected per strain (6 + 10). Data are summarized from two independent experiments. **(D)** Survival rate of mice infected with 10^4^ tachyzoites. In total, 14 mice were infected per strain (6 + 8). Data are summarized from two independent experiments. Statistical analysis was performed using a log rank test (*p* < 0.05). **(E)** Mice were infected via the intra-footpad route with 500 *T. gondii* tachyzoites of the RHΔ*gra14* mutant and its parental strain, and survival was monitored for 30 days. In total, 15 and 16 mice were infected per strain (RH, 7 + 8; RHΔ*gra14*, 8 + 8). **(F)** Mice were infected via the intra-footpad route with 500 *T. gondii* tachyzoites of the RHΔ*gra7* mutant and its parental strain, and survival was monitored for 30 days. In total, 8 and 8 mice were infected per strain (RH, 8; RHΔ*gra7*, 8). This experiment was performed once. Statistical analysis was performed using the log rank test (*p* < 0.05). *indicates a significant difference.

Next, we assessed the *in vivo* effects of each GRA on parasite virulence. Almost all mice intraperitoneally injected with 500 tachyzoites of the parental Pru parasites survived (15/16, 15/16, 12/14), whereas approximately 20% (3/16), 60% (10/16), and 0% (0/14) of mice survived after infection with PruΔ*gra7*, PruΔ*gra14*, and PruΔ*gra15* strains, respectively (Figures 7A–C). To further confirm the role of GRA14 in virulence, we infected mice by intraperitoneal injection with 10,000 tachyzoites of the parental Pru and PruΔ*gra14* parasites, and monitored mouse survival until 30 days post-infection. There was no significant difference in survival between mice infected with 10,000 parasites of the parental Pru and PruΔ*gra14* strains (Figure 7D).

Next, to determine the effect of GRA14 on *in vivo* parasite growth and the immune response at the site of infection, mice were infected with 500 tachyzoites of the parental Pru, PruΔ*gra14*, and GRA14-complemented lines. At 5 days after infection, mice were euthanized and the parasite burden and levels of cytokine secretion, including IL-12p40 and interferon-γ (IFN-γ), were examined. Mice infected with each strain showed no significant difference in parasite burden in the spleen or the peritoneal exudate cells (Supplemental Figures 7A,B). Although the differences in IL-12p40 and IFN-γ secretion by the peritoneal exudate cells were not significant, the average level of IFN-γ in PruΔ*gra14* mutant-infected mice was higher than that in the parental Pru and complemented strains (Supplemental Figures 7C,D). Moreover, no significant difference was detected in the level of serum IFN-γ on either day 3 or day 5 among these mouse groups (Supplemental Figures 7E,F).

To investigate how the deficiency of each GRA affects host immunity at an earlier time, we conducted a time-course experiment using thioglycolate-induced peritoneal macrophages (Supplemental Figure 8). Supernatants were collected every 6 h for 24 h and measured the production of IL-12p40. IL-12p40 production was abolished in the macrophages infected with PruΔ*gra7*, PruΔ*gra14*, and PruΔ*gra15* strains until 18 hours post-infection. However, the IL-12p40 production at 24 h post-infection decreased in the macrophages infected with the deficient parasite lines, with the highest decrease from PruΔ*gra15*, followed by PruΔ*gra7*, and then PruΔ*gra14*.

Lastly, we examined the effect of GRA7 and GRA14 on type I RH parasites. Mice were infected by intra-footpad injection with 500 parasites of the RHΔ*gra7*, RHΔ*gra14*, and parental RH parasite strains, respectively. Survival was monitored for 30 days and 86% (13/15) of mice infected with the parental RH strain survived, whereas all RHΔ*gra14* mutant-infected mice succumbed to the infection between 15 and 26 days after infection (Figure 7E). By contrast, when challenged with the parental RH and RHΔ*gra7* parasites, 0% (0/8) and 25% (2/8) of mice survived after infection with the parental RH and RHΔ*gra7* parasites, respectively (Figure 7F).

## Discussion

Secreted GRA15 has been identified as a major factor that contributes to the strain-specific differences in NFκB activation (15). Meanwhile, GRA7 produces a strong antibody response in the acute phase of infection (22) and has been tested as a candidate for vaccine development (23). Recent studies have revealed that GRA7 associates with ROP2 and ROP4, and functions in concert with ROP18 protein complexes that resist IFN-γ-activated host immune-related GTPase (24–26). Moreover, recombinant GRA7 interacts with inflammasome-related molecules, such as an apoptosis-associated speck-like protein that contains a caspase recruitment domain (ASC) and phospholipase D1 (PLD1) (27). However, few studies have investigated the role of GRA7 in the pathogenesis of type II *T. gondii* strains. In the present study, we demonstrated that GRA14 is involved with NFκB activation by *T. gondii*. GRA14 seems to be implicated in the interaction with host molecules because secreted GRA14 localizes to PVs containing membranous strand-like extensions (called PVM extensions) similar to other GRA proteins such as GRA3 and GRA7 (17). Furthermore, GRA14 is anchored in the PVM with its C terminus facing the host cell cytosol (17). GRA14 has also been reported as a potential vaccine candidate against *T. gondii* infection. Several studies have reported the protective immunity induced by vaccination with GRA14 antigen (28–32). However, there have been no previous reports regarding the modification of host cell function by GRA14. Thus, we targeted GRA7, GRA14, and GRA15 in this study.

Although we focused on NFκB signaling pathway, reporter activity by GRA was also evaluated in this study using reporter plasmids having response elements such as cAMP-responsive element, nuclear factor of activated T cells (NFAT), serum responsive element, serum responsive factor (SRF), and activated protein 1. As shown in Supplemental Figure 4, GRA14 and GRA15 activated all of them, while GRA7 activated NFAT and SRF other than NFκB. However, the main activities of GRAs were observed in NFκB activation. Interacting host factor of GRA14 is unknown, while GRA7 and GRA15 activate NFκB via TNF receptor-associated protein (TRAF). TRAF participates in the activation of the transcription factor NFκB and members of the mitogen-activated protein kinase (MAPK) family, including MAPK, c-jun N-terminal kinase, and p38. It remains possible that each GRA regulates host immunity via signaling pathway other than NFκB, but we believe that one of the primary sites of action is the NFκB pathway. Interestingly, although the levels of nuclear translocation of RelA in GRA7- and GRA14-expressing cells were significantly lower than in GRA15-expressing cells, expression alone was adequate for nuclear translocation. Moreover, cells infected with PruΔ*gra7* parasites showed no significant difference in the intensity of nuclear translocation compared with uninfected cells. In addition, GRA14 deficiency partially attenuated the intensity of nuclear RelA in cells infected with *T. gondii*. Collectively, these results suggest that GRA15 is the main player for NFκB activation by type II *T. gondii*. Additionally, GRA7 and GRA14 play a certain role in modulation of the NFκB pathway by type II *T. gondii*. By contrast, the levels of phosphorylated IκBα were comparable among cells infected with the parental Pru strain and mutant strains PruΔ*gra7* and PruΔ*gra14*. It was reported that GRA15-mediated NFκB activation was dependent on TRAF6, and GRA15 deficiency caused a decrease in the levels of phosphorylated-IκBα (15), which was consistent with our results. Contrary to this, another study showed that recombinant GRA7 also interacted with TRAF6, and recombinant GRA7 protein stimulated the phosphorylation of IκBα (16). However, in the present study, GRA7 deficiency showed no clear change in the phosphorylation level of IκBα. It may be that due to the higher activity of GRA15 compared with that of GRA7 and GRA14, GRA15 compensates for the loss of GRA7 and GRA14 function.

PruΔ*gra7*, PruΔ*gra14*, and PruΔ*gra15* strains induced significantly less cytokine secretion from infected macrophages than the parental Pru strain-infected cells. NFκB activation leads to the transcription of pro-inflammatory genes, such as those encoding IL-1β and IL-12 (15, 33). In addition, our transcriptome analysis revealed that these GRAs regulated the gene expression levels of similar inflammatory cytokines and chemokines by macrophages, in turn stimulating the development of a T-helper type 1 (Th1) immune response (33). Our data suggested that either GRA7 or GRA15 deficiency is sufficient for the increase in acute virulence in infected mice. Mice infected with a type II GRA15-deficient strain had a significantly higher parasite burden than mice infected with a parental type II strain (15). GRA15 activates NFκB in host cells and induces early IL-12 secretion (15). IL-12 stimulates NK cells and T cells to secrete IFN-γ (34). On day 2 after infection, mice infected with a type II GRA15-deficient strain had significantly less IFN-γ in their intraperitoneal cavities than mice infected with a parental type II strain (15). IFN-γ is the primary cytokine of host resistance to intracellular pathogens (35). Thus, this difference in IFN-γ levels was the likely cause of the virulence differences. It has been demonstrated that GRA7 interacts with TRAF6, inducing innate immune responses via the NFκB pathway in macrophages (16). Our results suggested that the GRA7-induced reporter activity of the NFκB promotor was less than that of GRA15. However, GRA7 also interacts with a number of host cell proteins, including ASC and PLD1, revealing a new facet of the role of GRA7 in the regulation of innate immune responses (36). Thus, GRA7 deficiency might result in increased mortality comparable to that of GRA15.

GRA14 deficiency also resulted in a slight but significant increase in virulence compared with the parental strain in mice after the injection of 500 parasites. Whereas, consistent with recent research involving 2 × 10^5^ parental type II Δ*gra14* parasites, such a difference was no longer detectable when 10,000 parasites were injected, which furthermore had the potential to cause lethal tissue damage (37). However, after 5 days of intraperitoneal infection, GRA14 did not affect the parasite burden or the level of cytokine secretion, including IL-12p40 and IFN-γ, from the peritoneal cavity. Moreover, no significant difference was detected in the levels of serum IFN-γ on days 3 or 5 among the groups of mice. The attenuated signal output caused by GRA14-deficiency may impair the proper immune response, resulting in an increased parasite burden in mice infected with PruΔ*gra14* parasites at an early stage (days 1–4), explaining the slight difference in virulence of this strain. The GRA-induced protective immune response against *T. gondii* in mice requires activation of antigen-presenting cells such as IL-12 production in the early stages of infection. If the parasites were controlled by the protective immune response in the early stage of infection, the level of the inflammatory marker IFN-γ would be suppressed. Therefore, the increased activity of PruΔ*gra14* at the initial stage of infection might increase IFN-γ level compared to the parental and complemented lines. Overall, our results suggest that GRA7 and GRA15 are the major contributors to *in vivo* virulence, whereas GRA14 has a relatively low impact on mice virulence. Furthermore, parasites deficient in GRA7 but not in GRA14 affect parasite growth *in vitro*. Moreover, a previous study reported that a type II Δ*gra15* mutant formed significantly larger plaques than a type II strain in HFF cells, but this was not apparent in mouse embryo fibroblast cells (15). These data indicate that growth differences in GRA7 and GRA15-deficient strains may affect their virulence in mice.

In this study, our experiments had focused on type II strains; however, we conjectured that the GRA14 proteins of type I strains are functional because there are few amino acid differences between the type I and II proteins (P43S, D323G, and S356V). The GRA15 proteins from type II and type III strains activate NFκB. Type II strains activate NFκB more strongly than type III strains, whereas the type I RH strain does not induce NFκB activation because it has a mutation in GRA15, leading to a frameshift and an early stop codon (15, 38). Therefore, because GRA15 of type I strains lacks activity, it is easy to evaluate the effect of GRA14 deficiency. Thus, we hypothesized that GRA14 might be involved with the mechanisms of NFκB activation by type I *T. gondii*. Previous studies have shown that type I strains interfere with the host NFκB pathway to promote their survival. ROP18, a key serine/threonine kinase that phosphorylates host proteins to modulate acute virulence, is associated with phosphorylation of RelA at Ser-468 and promotes the degradation of RelA to inhibit the NFκB pathway (39). Moreover, polymorphic kinase ROP16 of type I strains is capable of suppressing the IL-12 response of infected macrophages stimulated with lipopolysaccharide, thereby inhibiting NFκB transcriptional activity (15, 40). Whereas, other studies have shown that NFκB is activated by a type I strain of *T. gondii*, and that its activation is necessary for the inhibition of apoptosis (41–43). However, it is not known what effect GRAs have on the NFκB pathway.

Therefore, we lastly evaluated the effects of GRA7 and GRA14 deficiency in the type I RH strain on the survival of mice. Surprisingly, unlike type II parasites, all mice infected with RHΔ*gra14* parasites died within 26 days of footpad inoculation. Previous studies showed that GRA14 did not affect the growth and virulence of parasites following intraperitoneal injection of mice (17, 44). Unlike intraperitoneal inoculation, which results in a rapid, acute systemic infection, intra-footpad inoculation allows us to observe the gradual spread of *T. gondii in vivo* (45). Generally, intraperitoneal infection by RH tachyzoites was lethal. However, intra-footpad infection led to survival or, at least, a prolonged survival time in the present study. Therefore, deleting GRA14 may result in a lethal parasitic load in mice. By contrast, mice infected with RHΔ*gra7* parasites experienced a significant delay in death compared with the parental RH strain. A previous study reported that outbred CD-1 mice infected with RHΔ*gra7* parasites exhibited a similar phenotype (25). GRA7 binds to the GTP-bound immunity-related GTPase a6 and acts synergistically with ROP18 to block immunity-related GTPases (25, 26). Thus, these results suggest that GRA14 plays an important role in the control of parasite infection, creating a paradigm that protects the host animals from acute infection and death.

In conclusion, the present study demonstrated new molecular functions for GRA7 and GRA14 and confirmed their role in the induction of NFκB during a type II strain infection. NFκB activation mediated via GRA7, GRA14, and GRA15 was closely related to the Th1 response promoted by inflammatory cytokines following the activation of macrophages. This immune response limits the tissue invasion of the parasite, ensuring the survival of the host but, paradoxically, also aiding the survival of the parasite by converting it into a bradyzoite form able to persist in the muscle and brain tissues (46). GRA7 has multiple target components within the host cell that cause different virulence phenotypes dependent on the type of parasite. Whereas, the GRA14 protein has a low polymorphic phenotype and is potentially functional throughout type I, II, and III strains. Moreover, the suppressive control of virulence by early immune activation after infection, which has been regarded as a unique event to type II strains, is a conserved strategy across parasite strains. This may contribute to the high prevalence and wide distribution of this protozoan parasite. Thus, further insight into the precise role of these GRAs may help delineate the mechanism of NFκB modulation by *T. gondii*.

## Data Availability Statement

The original contributions presented in the study are publicly available. This data can be found in NCBI SRA: https://www.ncbi.nlm.nih.gov/sra/?term=DRA010408, accession number DRA010408.

## Ethics Statement

The experimental protocol was approved by the Committee on the Ethics of Animal Experiments at the Obihiro University of Agriculture and Veterinary Medicine (permit number: 19-50).

## Author Contributions

FI, RF, YH, KK, KU, ST, and RI conducted the experiments. FI, MY, and YN designed the experiments. FI and YN performed the data analyses and wrote the manuscript. All authors revised the manuscript and approved the final version.

## Conflict of Interest

The authors declare that the research was conducted in the absence of any commercial or financial relationships that could be construed as a potential conflict of interest.

## Supplementary Material

Supplemental Data Sheet 1 Detailed expression data for GRA-dependently regulated genes.

Supplemental Data Sheet 2 Results of KEGG pathway analysis.

Supplemental Data Sheet 3 Heat map.

Supplemental Figure 1 GRA knockout and the complementation strategies. **(A)** Schematic genomic representation of the GRA7 locus and the plasmid construct used to target the GRA7 gene. The drug-resistant hypoxanthine-xanthine-guanine phosphoribosyl transferase (HXGPRT) cassette was surrounded by the 5′ and 3′ untranslated regions (UTR) of GRA7. **(B)** Schematic representation of the CRISPR/CAS9 strategy used to inactivate the target genes by inserting the pyrimethamine-resistance DHFR cassette (DHFR^*^). Transfection of the CRISPR plasmid targeting TgGRA (G01), together with an amplicon containing the DHFR^*^-expressing cassette flanked by regions homologous to the target gene, was used to disrupt the corresponding target gene by insertion. **(C)** Transfection of the CRISPR plasmid targeting the TgUPRT gene, together with an amplicon containing 1,000 by of the 5′ and 3′ untranslated regions (5′ UTR and 3′ UTR) flanked by regions homologous to the TgUPRT gene, was used.

Supplemental Figure 2 IFAT and western blotting to confirm the expression of TgGRA7 and TgGRA14. **(A)** IFAT analysis of Vero cells infected with Pm, PruAgra7 (deltaGRA7), PruAgra14 (deltaGRA14), and complemented (CompGRA7 and CompGRA14) parasites at 24 h post-infection. Cells were fixed and stained with a-TgSAG1 (green), a-TgGRA7 (red), a-TgGRA14 (red), and Hoechst dye (blue). Bars, 10 gm. **(B)** Western blots of the parasite strains. Into each lane, 1 × 10^6^ parasites were loaded. Anti-TgGRA7 and anti-GRA14 antibodies detected 25.9 and 42 kDa proteins in the parental Pru and complemented parasites, respectively, but not in the deficient mutant parasites.

Supplemental Figure 3 Forced expression of GRA7, GRA14, and GRA15 in 293T cells. 293T cells were transiently transfected with the expression vectors for GRA7, GRA14, and GRA15. Cells lysates were then separated by SDS-PAGE, and western blot analysis was carried out using an anti-FLAG antibody. The estimated sizes of the FLAG-tag fused to GRA7, GRA14, and GRA15 were 29.9, 48.7, and 61.8 kDa, respectively. However, the observed molecular weight of the FLAG-tag fused to GRA15 (−75 kDa), was higher than the expected predicted size (61.8 kDa). It has been shown that this is not caused by parasite-mediated modification of GRA15. Most likely, it is the particular amino acid composition of GRA15, which is enriched in Pro, Ser, and Thr, that makes it run slower than expected on an SDS-PAGE gel. Black arrows indicate the estimated band sizes of the target proteins.

Supplemental Figure 4 Luciferase activities in 293T cells transfected with various reporter plasmids. 293T cells were transiently transfected with pGL4.29 (CRE), pGL4.30 (NFAT), pGL4.32 (NFx13), pGL4.33 (SRE), pGL4.34 (SRF), and pGL4.44 (AP1) expressing firefly luciferase and pGL4.74 expressing renilla luciferase. Cells were immediately transfected with the expression vectors of GRA7, GRA14, GRA15, and the empty p3 × FLAG-cmv14 vector used as a negative (empty) control. The promoter activity was shown as relative fold-increase as compared to control cells in the luciferase activity normalized for Renilla luciferase activity. Values are the means of triplicate samples.

Supplemental Figure 5 Infection rate, growth, and egress assay. **(A)** Infection rates of the different parasite lines in Vero cells at 24 h post-infection. **(B)** Egress rates of the different parasite lines in Vero cells at 72 h post-infection. **(C)** Intracellular replication assay of the parasite lines in Vero cells at 48 h post-infection. Each bar represents the means ± the standard deviation (*n* = 4 for all groups), and the results represent two independent experiments with similar results. Statistical analysis was performed using one-way ANOVA, ^*^a significant difference (*p* < 0.05).

Supplemental Figure 6 Expression levels of chemokines and cytokines in Raw246.7 macrophage cells. **(A,B)** Raw246.7 macrophage cells were infected with parasite strains for 24 h, then cells were lysed, and total RNA was extracted. RNA was used to synthesize cDNA. Real-time RT-qPCR amplification was carried out for CXCL1, CXCL5, IL-lbeta, IL-6, and Cc117. Each bar represents the mean ± the standard deviation (*n* = 3 for all groups), and the results are from a single experiment. Statistical analysis was performed using one-way ANOVA with *post-hoc* Tukey's test, ^*^a significant difference (*p* < 0.05).

Supplemental Figure 7 *In vivo* cytokine ELISA and parasite burden. Mice were infected with 500 tachyzoites of the parasites and then euthanized to examine the parasite burden and level of cytokine secretion, including IL-12p40 and IFN-y, 5 days after infection. **(A,B)** Parasite burden in the spleen and peritoneal cavity in mice infected with parental Pru, PruLgra14 (deltaGRA14), and the complemented parasites (Comp). **(C,D)** Levels of serum IFN-y and IL-12p40 in mice. **(E,F)** Cytokine levels in the peritoneal cavity in mice. Each plot represents data from one mouse. Statistically significant differences were analyzed by one-way ANOVA with *post-hoc* Tukey's test but no significant difference was found. Data were collected from one experiment.

Supplemental Figure 8 Levels of interleukin-12p40 in thioglycolate-elicited macrophage cells. Macrophages were infected with the parental Pru, PruAgra7, PruAgra14, and PruAgral5 parasite strains. At every 6 h for 24 h post-infection, supernatants were collected, and IL-12p40 levels were determined by cytokine ELISA. Values are the means ± SD of four samples, ^*^a significant difference (*p* < 0.05). #a significantly lower level of IL-12p40 compared with the Pm strain infected cells (*p* < 0.05). Differences were tested by one-way ANOVA with Turkey's *post-hoc* test.
