# Supplementary figures and images for: Toxoplasma gondii Dense Granule Proteins 7, 14, and 15 Are Involved in Modification and Control of the Immune Response Mediated via NF-κB Pathway
Source: Front Immunol. 2020 Jul 31;11:1709. doi: 10.3389/fimmu.2020.01709 (PMC7412995; doi:10.3389/fimmu.2020.01709)

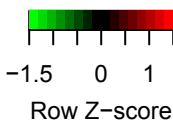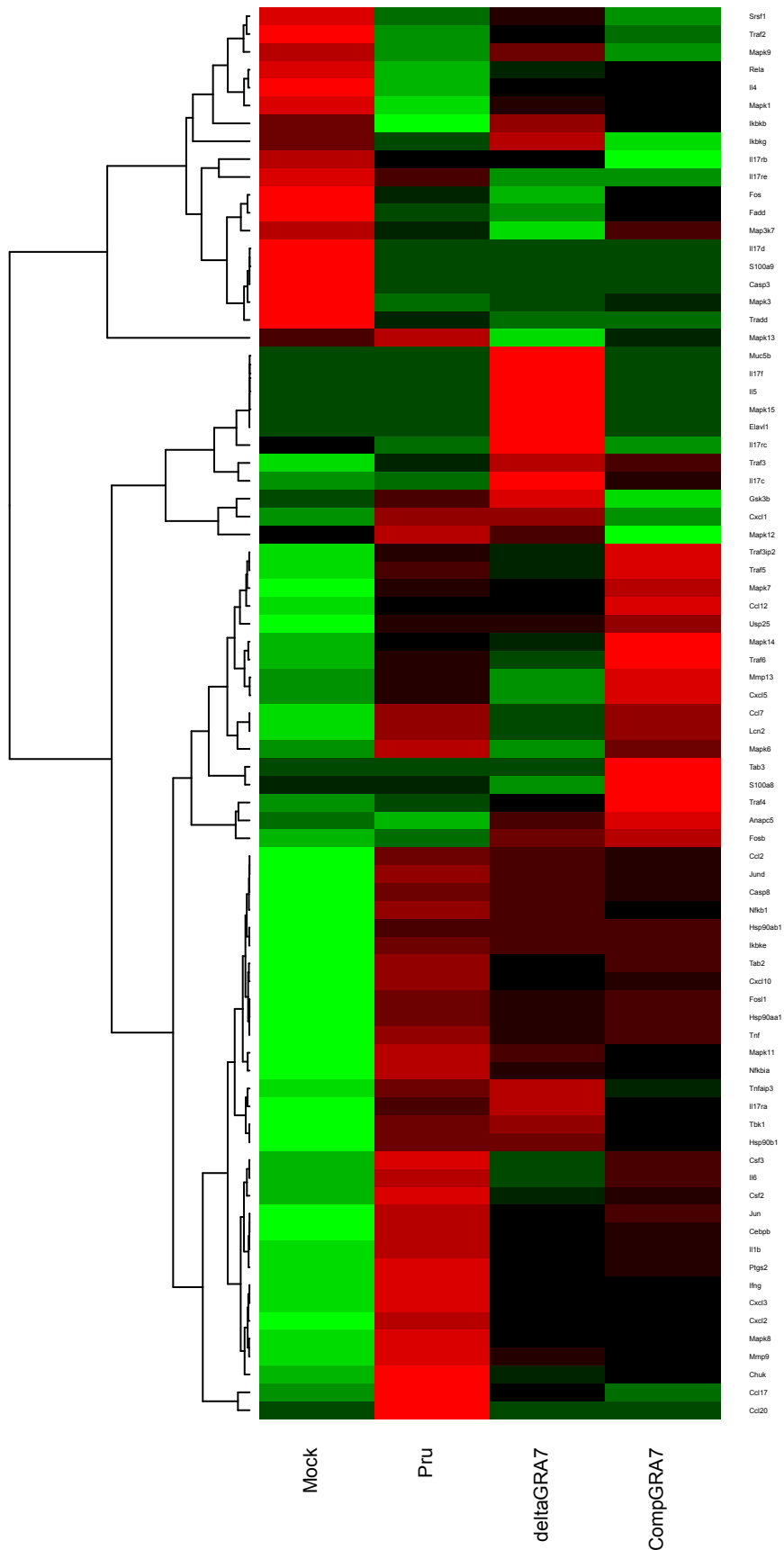

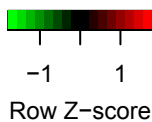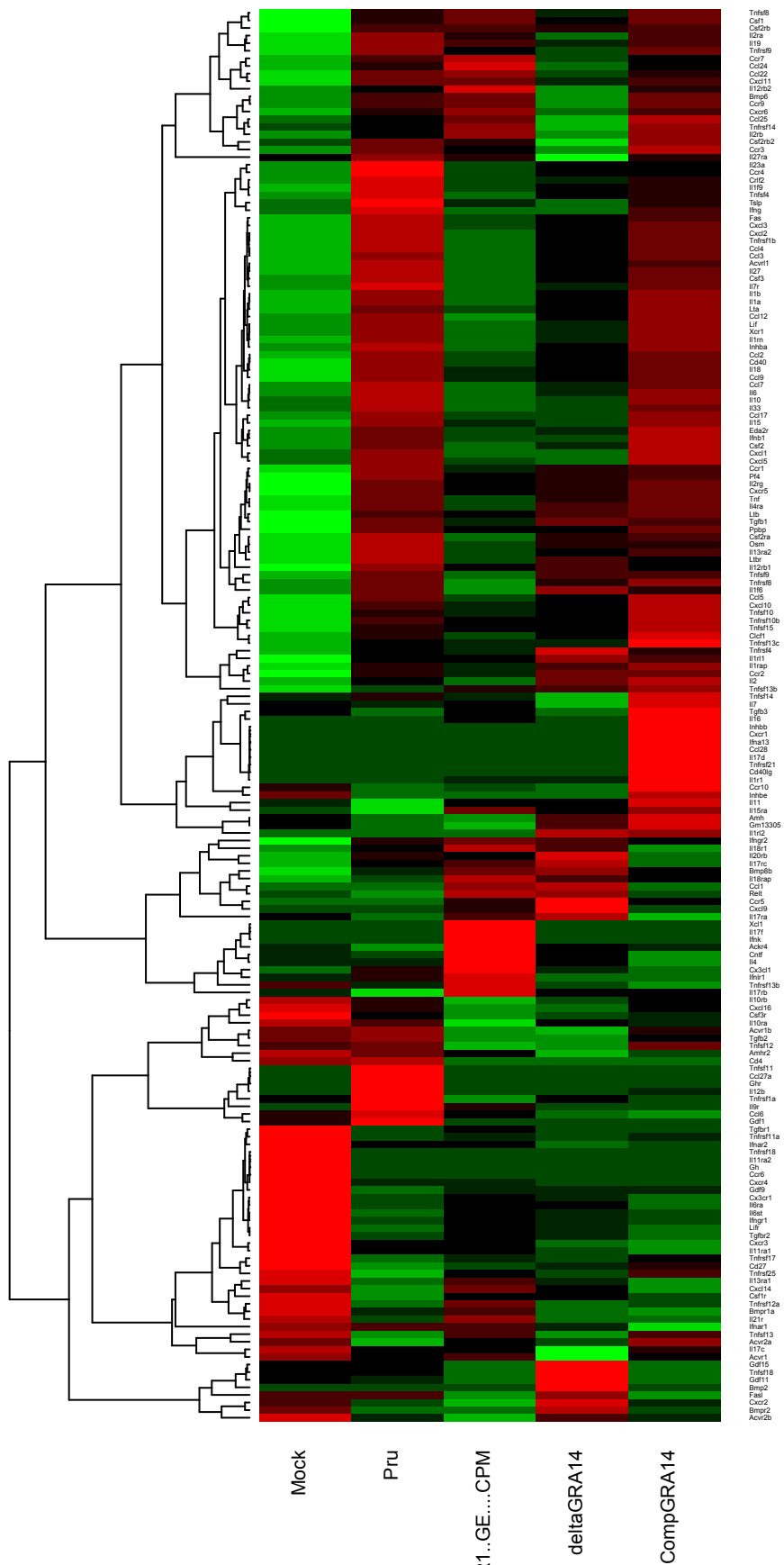

Supplement: Supplemental Data Sheet 3 — Heat map. [file Data_Sheet_3.pdf]

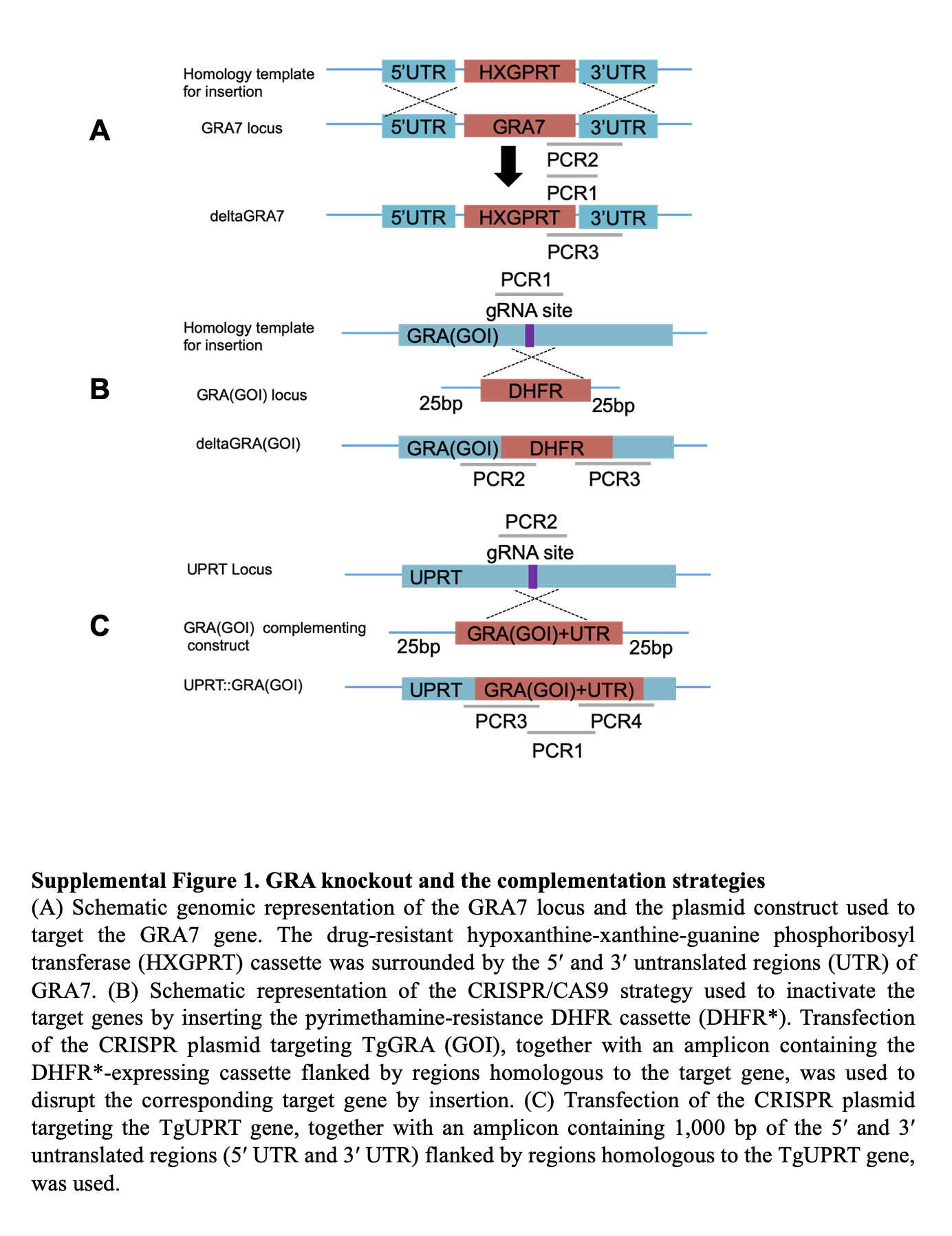

Supplement: Supplemental Figure 1 — GRA knockout and the complementation strategies. (A) Schematic genomic representation of the GRA7 locus and the plasmid construct used to target the GRA7 gene. The drug-resistant hypoxanthine-xanthine-guanine phosphoribosyl transferase (HXGPRT) cassette was surrounded by the 5′ and 3′ untranslated regions (UTR) of GRA7. (B) Schematic representation of the CRISPR/CAS9 strategy used to inactivate the target genes by inserting the pyrimethamine-resistance DHFR cassette (DHFR*). Transfection of the CRISPR plasmid targeting TgGRA (G01), together with an amplicon containing the DHFR*-expressing cassette flanked by regions homologous to the target gene, was used to disrupt the corresponding target gene by insertion. (C) Transfection of the CRISPR plasmid targeting the TgUPRT gene, together with an amplicon containing 1,000 by of the 5′ and 3′ untranslated regions (5′ UTR and 3′ UTR) flanked by regions homologous to the TgUPRT gene, was used. [file Image_1.TIF]

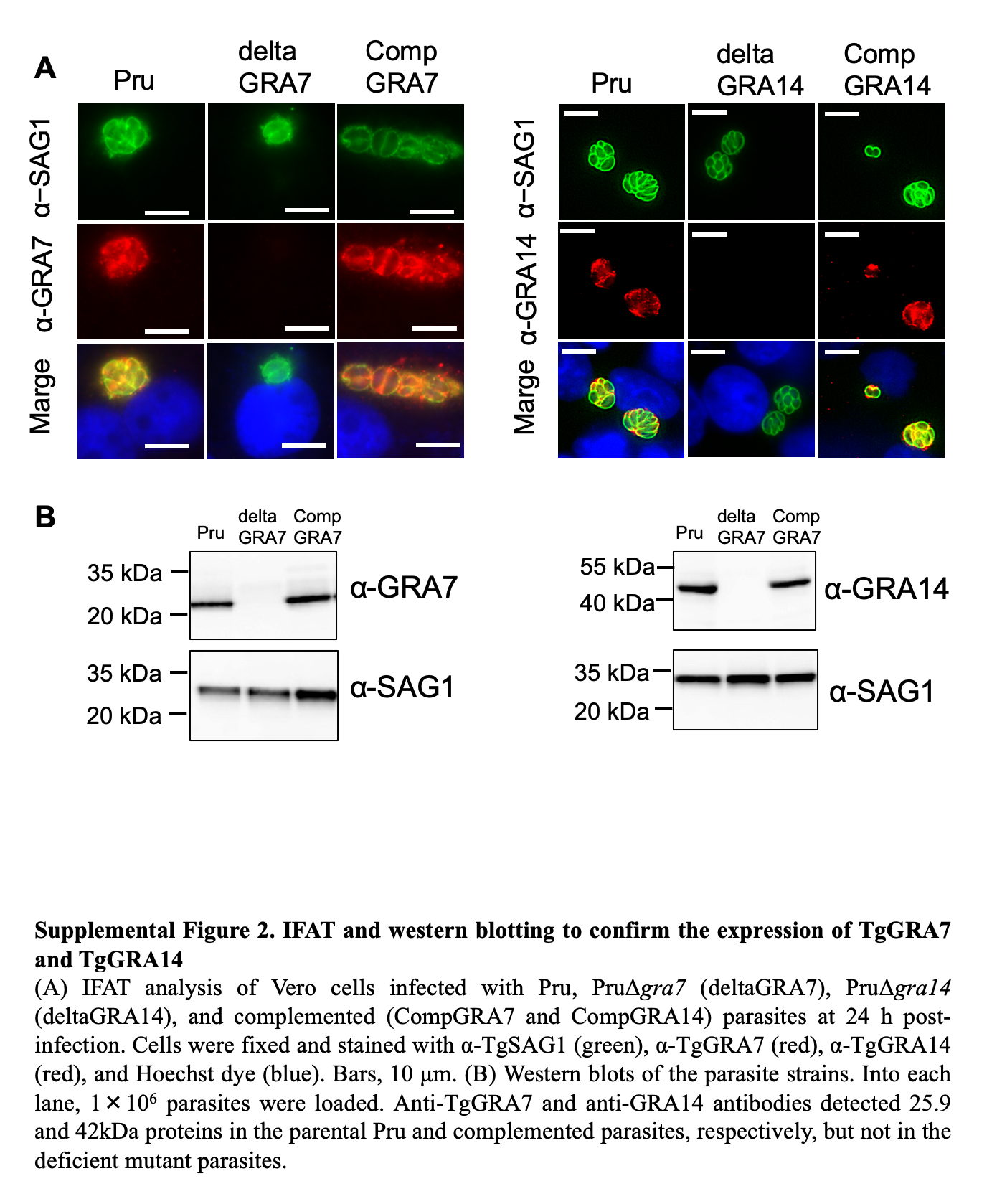

Supplement: Supplemental Figure 2 — IFAT and western blotting to confirm the expression of TgGRA7 and TgGRA14. (A) IFAT analysis of Vero cells infected with Pm, PruAgra7 (deltaGRA7), PruAgra14 (deltaGRA14), and complemented (CompGRA7 and CompGRA14) parasites at 24 h post-infection. Cells were fixed and stained with a-TgSAG1 (green), a-TgGRA7 (red), a-TgGRA14 (red), and Hoechst dye (blue). Bars, 10 gm. (B) Western blots of the parasite strains. Into each lane, 1 × 10^6^ parasites were loaded. Anti-TgGRA7 and anti-GRA14 antibodies detected 25.9 and 42 kDa proteins in the parental Pru and complemented parasites, respectively, but not in the deficient mutant parasites. [file Image_2.TIF]

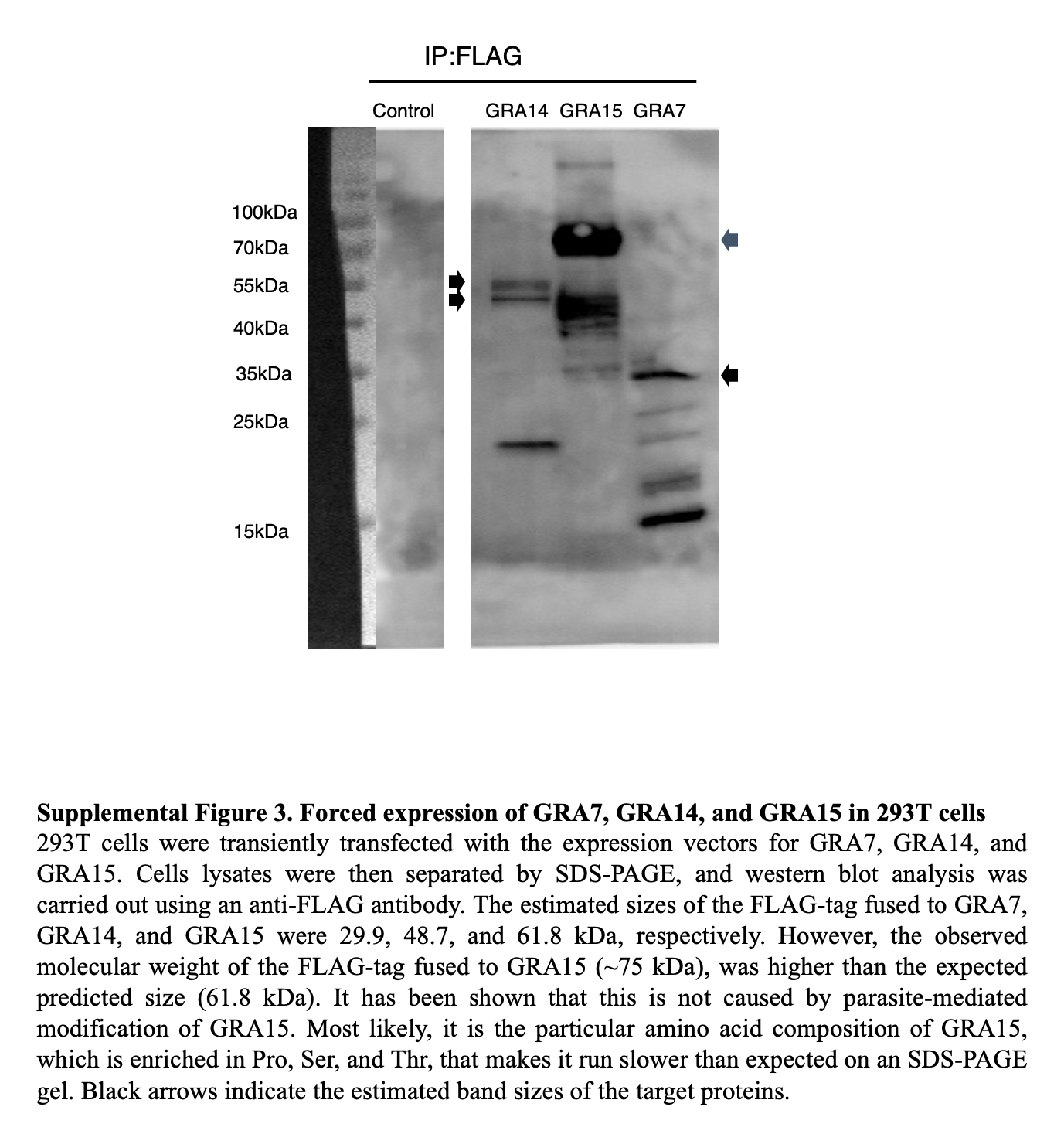

Supplement: Supplemental Figure 3 — Forced expression of GRA7, GRA14, and GRA15 in 293T cells. 293T cells were transiently transfected with the expression vectors for GRA7, GRA14, and GRA15. Cells lysates were then separated by SDS-PAGE, and western blot analysis was carried out using an anti-FLAG antibody. The estimated sizes of the FLAG-tag fused to GRA7, GRA14, and GRA15 were 29.9, 48.7, and 61.8 kDa, respectively. However, the observed molecular weight of the FLAG-tag fused to GRA15 (−75 kDa), was higher than the expected predicted size (61.8 kDa). It has been shown that this is not caused by parasite-mediated modification of GRA15. Most likely, it is the particular amino acid composition of GRA15, which is enriched in Pro, Ser, and Thr, that makes it run slower than expected on an SDS-PAGE gel. Black arrows indicate the estimated band sizes of the target proteins. [file Image_3.TIF]

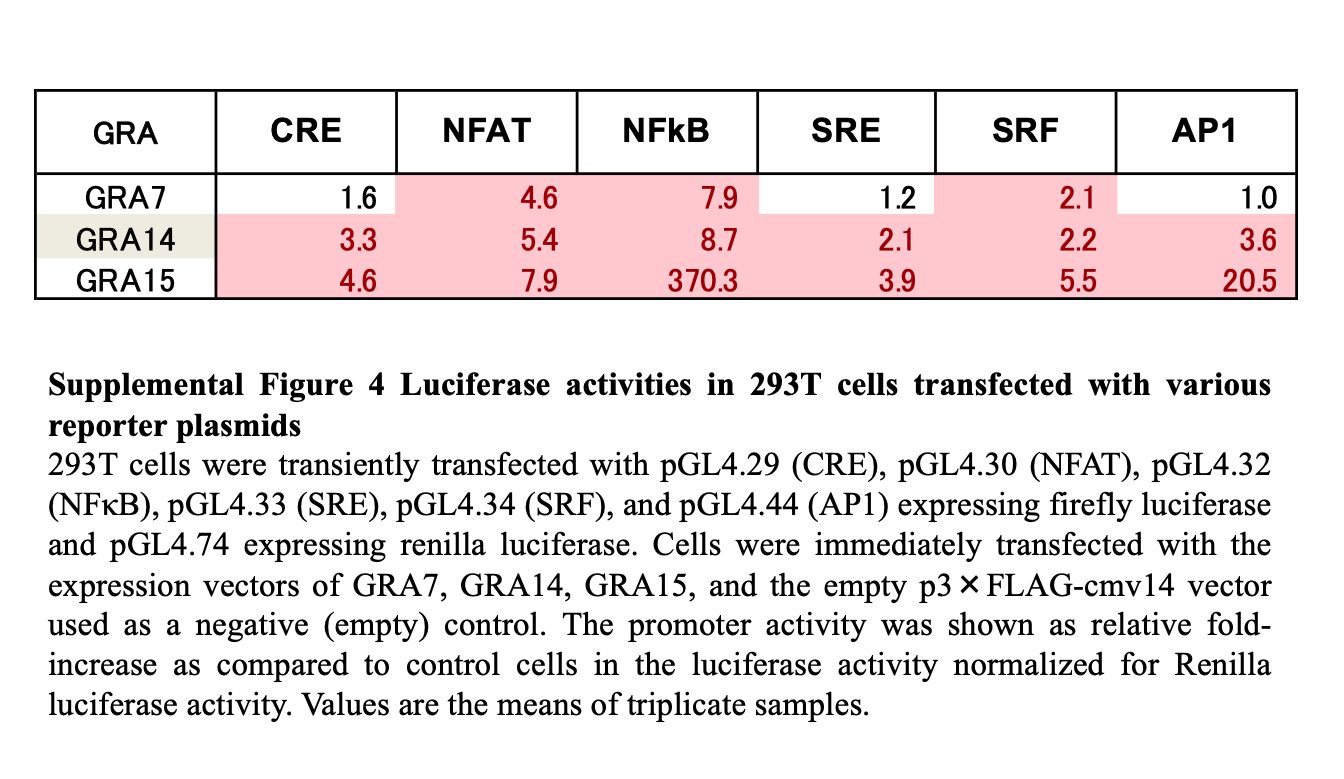

Supplement: Supplemental Figure 4 — Luciferase activities in 293T cells transfected with various reporter plasmids. 293T cells were transiently transfected with pGL4.29 (CRE), pGL4.30 (NFAT), pGL4.32 (NFx13), pGL4.33 (SRE), pGL4.34 (SRF), and pGL4.44 (AP1) expressing firefly luciferase and pGL4.74 expressing renilla luciferase. Cells were immediately transfected with the expression vectors of GRA7, GRA14, GRA15, and the empty p3 × FLAG-cmv14 vector used as a negative (empty) control. The promoter activity was shown as relative fold-increase as compared to control cells in the luciferase activity normalized for Renilla luciferase activity. Values are the means of triplicate samples. [file Image_4.TIF]

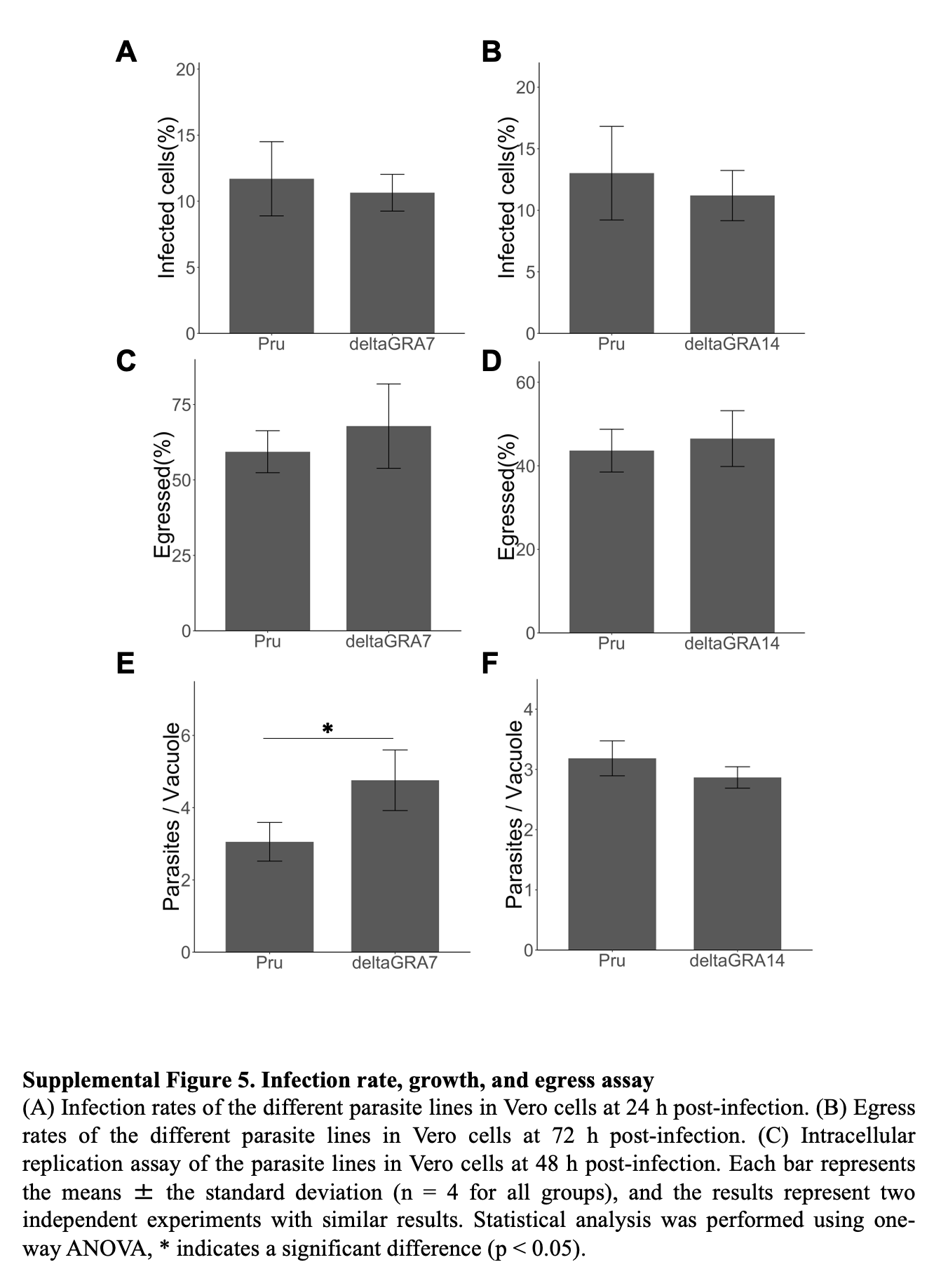

Supplement: Supplemental Figure 5 — Infection rate, growth, and egress assay. (A) Infection rates of the different parasite lines in Vero cells at 24 h post-infection. (B) Egress rates of the different parasite lines in Vero cells at 72 h post-infection. (C) Intracellular replication assay of the parasite lines in Vero cells at 48 h post-infection. Each bar represents the means ± the standard deviation (n = 4 for all groups), and the results represent two independent experiments with similar results. Statistical analysis was performed using one-way ANOVA, *a significant difference (p < 0.05). [file Image_5.TIF]

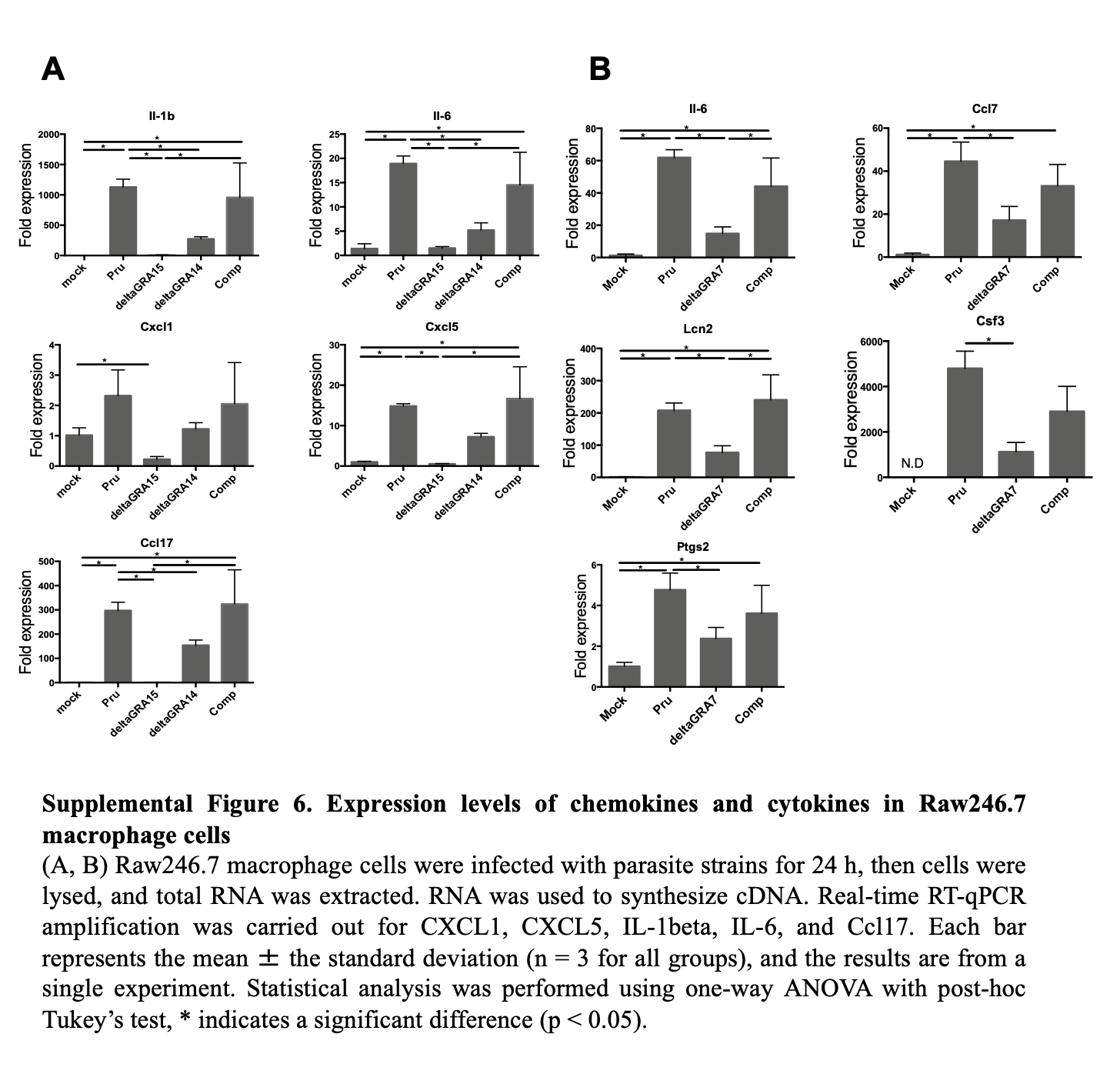

Supplement: Supplemental Figure 6 — Expression levels of chemokines and cytokines in Raw246.7 macrophage cells. (A,B) Raw246.7 macrophage cells were infected with parasite strains for 24 h, then cells were lysed, and total RNA was extracted. RNA was used to synthesize cDNA. Real-time RT-qPCR amplification was carried out for CXCL1, CXCL5, IL-lbeta, IL-6, and Cc117. Each bar represents the mean ± the standard deviation (n = 3 for all groups), and the results are from a single experiment. Statistical analysis was performed using one-way ANOVA with post-hoc Tukey's test, *a significant difference (p < 0.05). [file Image_6.TIF]

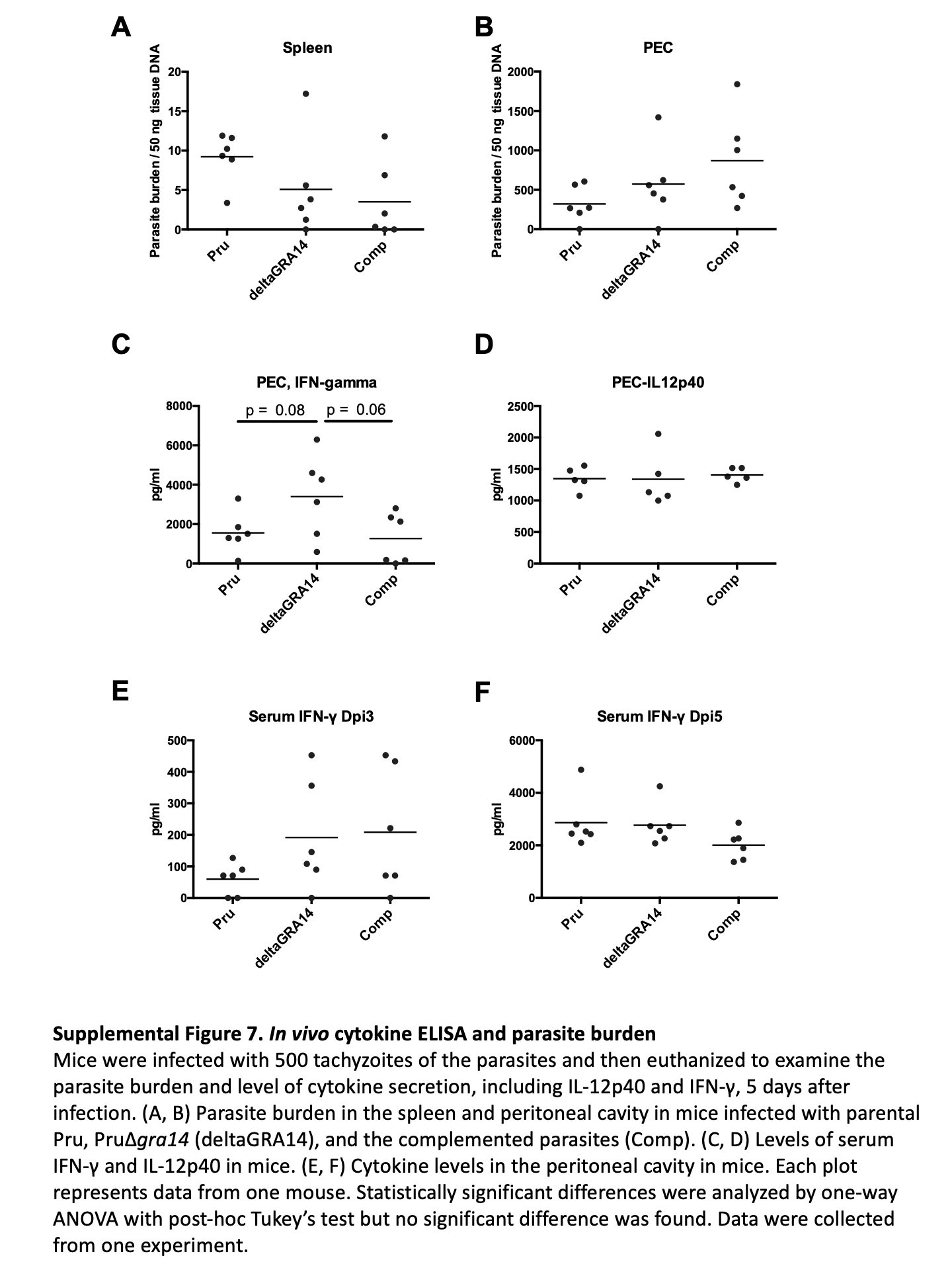

Supplement: Supplemental Figure 7 — In vivo cytokine ELISA and parasite burden. Mice were infected with 500 tachyzoites of the parasites and then euthanized to examine the parasite burden and level of cytokine secretion, including IL-12p40 and IFN-y, 5 days after infection. (A,B) Parasite burden in the spleen and peritoneal cavity in mice infected with parental Pru, PruLgra14 (deltaGRA14), and the complemented parasites (Comp). (C,D) Levels of serum IFN-y and IL-12p40 in mice. (E,F) Cytokine levels in the peritoneal cavity in mice. Each plot represents data from one mouse. Statistically significant differences were analyzed by one-way ANOVA with post-hoc Tukey's test but no significant difference was found. Data were collected from one experiment. [file Image_7.TIF]

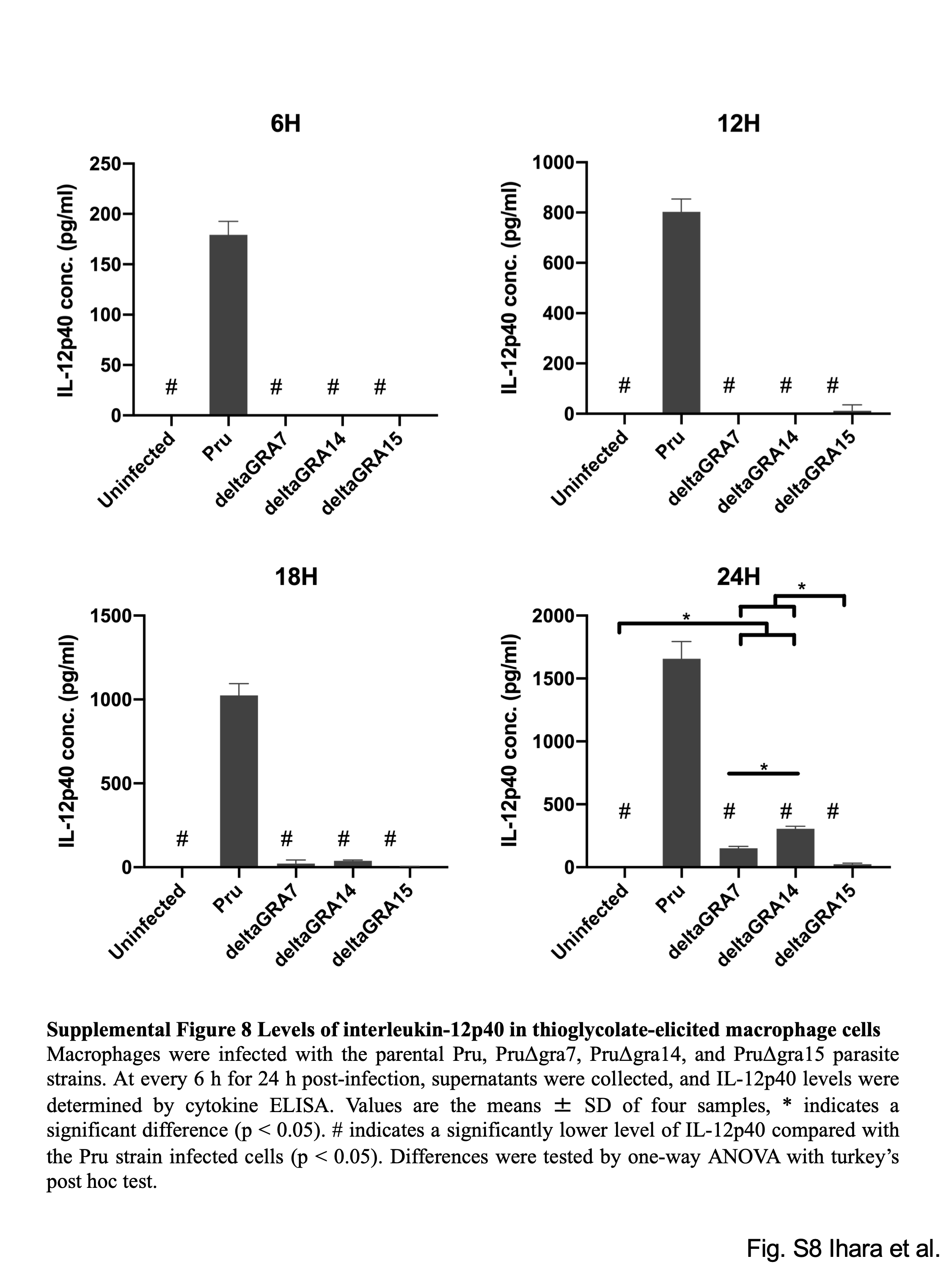

Supplement: Supplemental Figure 8 — Levels of interleukin-12p40 in thioglycolate-elicited macrophage cells. Macrophages were infected with the parental Pru, PruAgra7, PruAgra14, and PruAgral5 parasite strains. At every 6 h for 24 h post-infection, supernatants were collected, and IL-12p40 levels were determined by cytokine ELISA. Values are the means ± SD of four samples, *a significant difference (p < 0.05). #a significantly lower level of IL-12p40 compared with the Pm strain infected cells (p < 0.05). Differences were tested by one-way ANOVA with Turkey's post-hoc test. [file Image_8.TIFF]
